# How Neuromorphic Microstructures Control In Vitro Early‐Stage Neuronal Outgrowth

**DOI:** 10.1002/advs.202510822

**Published:** 2026-05-19

**Authors:** Claudia Latte Bovio, Esther Matamoros, Valentina Mollo, Anna Mariano, Valeria Criscuolo, Francesca Santoro

**Affiliations:** ^1^ Tissue Electronics Istituto Italiano di Tecnologia Naples Italy; ^2^ Dipartimento di Chimica, Materiali e Produzione Industriale Università di Napoli Federico II Naples Italy; ^3^ Neuroelectronic Interfaces, Faculty of Electrical Engineering and IT RWTH Aachen Aachen Germany; ^4^ Institute for Biological Information Processing‐Bioelectronics (IBI‐3) Forschungszentrum Juelich Juelich Germany

**Keywords:** artificial spines, early‐stage neuronal interfacing, neuromorphic biomaterials, neuronal polarity and guidance, two‐photon polymerization

## Abstract

Neuromorphic biomaterials represent a novel class of materials designed to replicate the architecture and functionality of neuronal structures, offering new opportunities in tissue engineering and bioelectronics. By mimicking the complex microenvironment of native neural tissue, biomimetic microstructures provide physical scaffolding to support and guide neuronal processes, with potential applications in chip‐based platforms for monitoring and stimulating neuronal networks. However, achieving precise control over the morphology of neuromorphic materials and understanding their influence on early‐stage neuronal development are remaining challenges. In this study, we present biomimetic microstructure arrays, fabricated via two‐photon polymerization, that emulate the diverse morphologies and spatial arrangements of dendritic spines. These structures enable the investigation of neuronal responses at the early developmental stage, focusing on key processes such as cell adhesion, neuronal polarity, growth cone dynamics, and network formation.

## Graphical abstract

Biomimetic approach to neuronal interaction with neuromorphic microstructures.

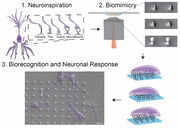

## 1 Introduction

Neuromorphic biomaterials are engineered to mimic the complex architecture and functionality of the nervous system, offering new avenues for studying, interfacing with, and potentially repairing neural tissues [1, 2]. By emulating the properties of neuronal networks, such as their topology, electrical conductivity, and dynamic responses, neuromorphic biomaterials provide a more physiological environment for neuronal growth and development [3, 4]. For example, these materials could be used to create more biocompatible interfaces to trigger biorecognition and on‐chip integration [5, 6]. In this scenario, the design of neuromorphic biomaterials often involves creating micro‐ and nanoscale structures that closely resemble the intricate features of neuronal cells, including dendrites, axons, and synaptic connections, and have shown great potential in regulating cell fate and have been exploited for bioelectronic applications [5, 7, 8, 9, 10]. These structures provide physical scaffolding to support and guide neuronal processes [11, 12, 13], promoting the formation of functional neural circuits [14, 15]. Moreover, these materials can dynamically respond to biological signals, such as changes in electrical activity or chemical gradients [16, 17, 18], further enhancing their integration with living tissues [19, 20, 21, 22]. Understanding neuronal responses at early developmental stages, when coupled to artificial neuromorphic materials, is crucial as they can trigger and modulate critical processes such as axonal outgrowth, growth cone navigation, and synapse formation, which are essential for establishing functional neural networks [3]. Investigating how neurons interact with biomaterials at these stages provides insight into key developmental mechanisms, including neuronal polarization, adhesion processes, and cytoskeletal dynamics [3, 23, 24]. Furthermore, early‐stage neuronal responses can leverage the design of biomaterials that optimize adhesion, differentiation, and guided neurite extension, which are fundamental in various applications such as neuroprosthetics, brain‐machine interfaces [25, 26, 27], and tissue regeneration [11, 28, 29]. Furthermore, important neuronal structures both at the structural and functional level are dendritic spines, specialized protrusions on neuronal dendrites [30].

Here, dendritic spines can be classified into several subtypes from a morphological point of view, including stubby, thin, and mushroom‐shaped spines, each reflecting distinct functional and developmental stages [3, 31]. Their structure, defined by parameters such as head size, neck diameter, and length, directly affects synaptic properties [32, 33, 34]. For instance, larger spine heads correspond to increased postsynaptic density (PSD) areas and greater receptor densities, promoting stronger synaptic transmission [35]. Thin spines, in contrast, are dynamic and adapt rapidly to changing synaptic inputs, making them central to synaptic plasticity. These variations in spine morphology enable neurons to finely tune synaptic responses, underscoring their significance in neural network connectivity [3, 36, 37, 38].

Beyond morphology, spine density is another critical factor influencing synaptic connectivity and neuronal function [39, 40]. In fact, dendritic spine density is highly variable, depending on developmental stage, brain region, and functional activity. Higher spine densities are often associated with enhanced synaptic integration, whereas lower densities may reflect synaptic pruning or adaptive plasticity [36, 41, 42].

Inspired by these considerations, a significant challenge in the development of neuromorphic biomaterials is achieving precise control over morphology and properties to effectively mimic the physiological environment of neural tissue. Previous studies have demonstrated that nanoscale topographies can significantly modulate neuronal development by altering physical cues at the cell–substrate interface [10, 21, 38, 43, 44]. For instance, size‐modulated nanopillar arrays have been shown to influence neuronal morphology, promote neurite elongation, and enhance synapse formation [25, 45, 46]. Similarly, bioinspired micro‐ and nanostructured neural interfaces have replicated some aspects of the extracellular matrix, guiding axonal alignment and supporting neuronal maturation through controlled geometry and material stiffness [3, 46, 47, 48, 49]. More recently, 3D‐printed nanostructured arrays fabricated via laser‐assisted methods have revealed that variations in the effective shear modulus of the substrate can direct network connectivity and influence growth cone morphology, suggesting a mechanical component in topographical guidance [24, 25, 50].

Despite these advances, most engineered platforms still lack the morphological specificity needed to mimic dendritic spines, structures that vary widely in shape (e.g., mushroom, thin, stubby) and play a key role in synaptic plasticity. Moreover, the combinatorial effects of different nanofeature shapes and spatial densities on neuronal behavior remain insufficiently explored. Understanding how these parameters interact to influence confinement, polarity, and synaptogenesis is crucial for the development of next‐generation biomimetic neural interfaces [9, 13, 23, 25, 37, 50, 51, 52].

Our study addresses these gaps by introducing a biomimetic platform inspired by dendritic spine morphologies, including thin, mushroom‐shaped, and stubby, which represent distinct stages of spinogenesis and synaptic development. These structures were fabricated using two‐photon polymerization (2PP), a technique that enables high‐precision manufacturing of complex geometries with customizable spatial arrangements [29, 53, 54]. This flexibility surpasses the limitations of previous fabrication methods and allows the exploration of the relationship between shape, density, and neuronal behavior. A key innovation of this work is its focus on how microstructure density and shape affect neuronal responses, particularly in relation to the degree of physical confinement. Furthermore, we aim to assess the effects of biomimetic microstructures on neuronal cytoskeleton architecture, growth cone dynamics, and network formation. Through advanced imaging techniques such as focused ion beam‐assisted milling and scanning electron microscopy (FIB‐SEM) [12, 55, 56], we reconstructed 3D interactions at the cell‐material interface, highlighting membrane deformations and protein localization. By integrating biomimetic design, precision fabrication, and comprehensive biological analysis, this work advances our understanding of neuron‐material interactions and lays the groundwork for developing more effective neuromorphic interfaces for neuroengineering and therapeutic applications.

## 2 Results and Discussion

**FIGURE 1:**
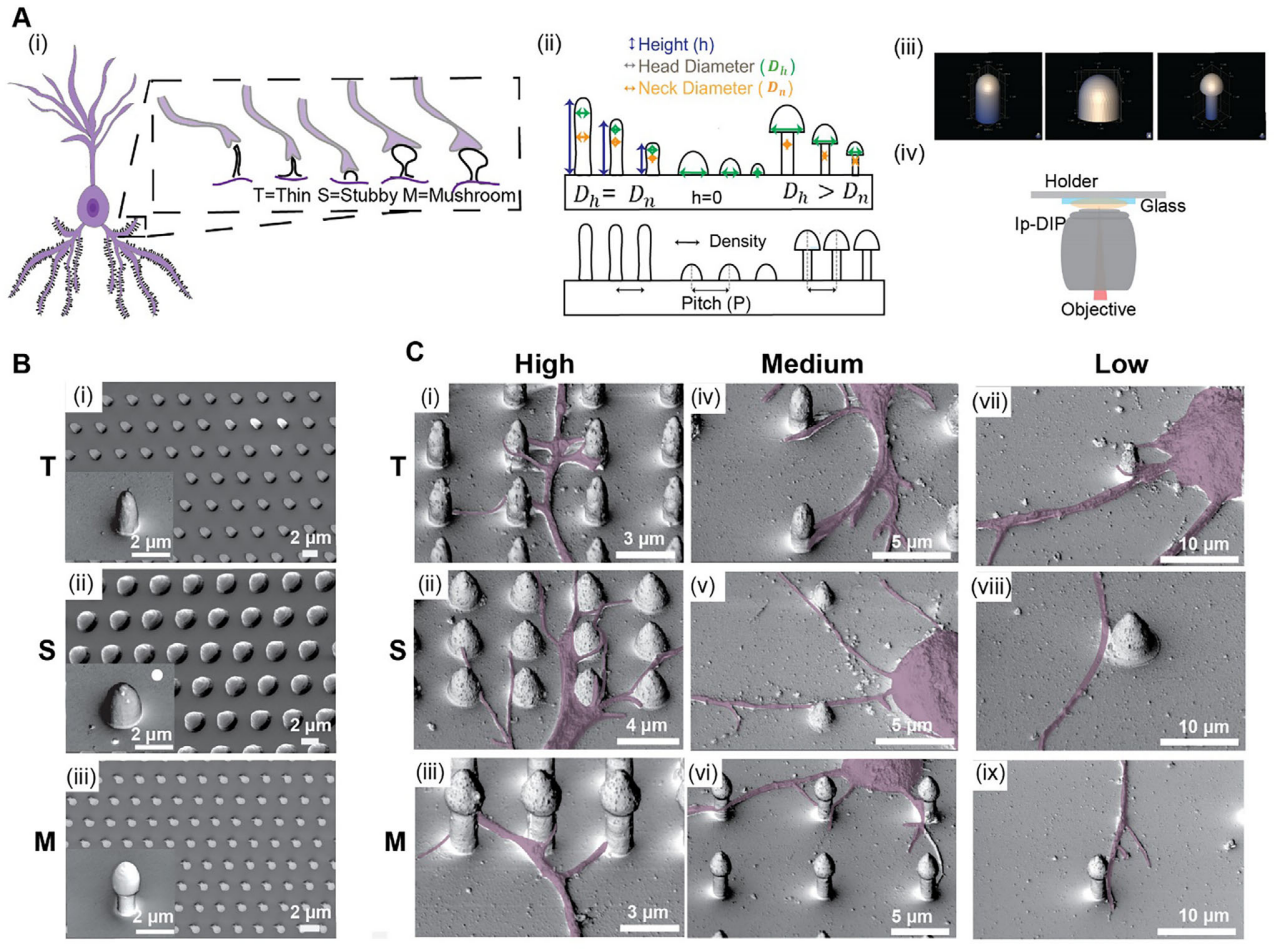
Biomimetic artificial spines design and early‐stage effect on neuronal outgrowth. (A) Spine geometry design taken from (i) living dendritic shapes T = thin‐shaped, S = stubby‐shaped, and M = mushroom‐shaped. (ii) design parameters: height (h), head diameter (D_h_), neck diameter (D_n_). The T shows D_n_ = D_h_, the S has h = 0, and M has D_h_ > D_n_. (iii) Artificial spine design via AutoCAD and (iv) fabrication process via 2PP. (B) Scanning Electron Microscopy (SEM) images of three microstructured high density arrays (i) T‐Shaped, (ii) S‐Shaped, (iii) M‐Shaped. (C) Pitch were chosen to achieve high‐density spine arrays (0.28 spines/µm^2^) (i) T, (ii) S, (iii) M, medium‐density spine arrays (0.11 spines/µm^2^) (iv) T, (v) S, (vi) M and low‐density arrays (0.045 spines/µm^2^) (vii) T, (viii) S, (ix) M.

Drawing inspiration from the diversity of dendritic spine morphologies observed in living neurons (Figure 1A(i)), we designed three corresponding artificial spine types (Figure 1A(ii)): thin‐shaped (T), associated with early synaptogenesis; mushroom‐shaped (M), linked to stable, mature synapses; and stubby‐shaped (S), which contribute to synaptic stability and serve as transitional forms during neurodevelopment. To reflect the physiological variability in spine morphology, we varied the geometry of the artificial spines by adjusting their height (h), neck diameter (D_n_), and head diameter (D_h_) (Figure 1A(iii); Figure S1 and Table S1).

High‐resolution fabrication was achieved via two‐photon polymerization (2PP) (Figure 1A(iv), Experimental Section) and scanning electron micrographs (SEM) of the fabricated structures are presented in Figure 1B (additional micrographs in Figures S2–S4), illustrating the uniform distribution and distinct morphological profiles of each spine‐mimetic microstructure array. Specifically, the arrays consist of regularly spaced pillars with well‐defined geometries corresponding to the thin‐shaped (Figure 1B(i)), stubby‐shaped (Figure 1B(ii)), and mushroom‐shaped (Figure 1B(iii)) designs, thanks to the 2PP fabrication process that allowed for the design of shape‐specific features such as neck constriction and head enlargement (Figures S2–S4) First, we investigated how spine density influences neuronal behavior, by engineering three distinct configurations considering the spine density as the number of structures per unit area (Figure 1C), with high (0.28 spines/µm^2^) (Figure 1C(i)–(iii)), medium (0.11 spines/µm^2^) (Figure 1C(iv)–(vi)), and low (0.045 spines/µm^2^) density configurations (Figure 1C(vii)–(ix)). This range allowed to examine how varying degrees of topographical confinement might affect neuronal outgrowth, adhesion, and network formation.

Following fabrication, the structures were functionalized with poly‐L‐lysine, and primary cortical neurons (Experimental Section) were cultured on the substrates to assess cellular responses at various stages of outgrowth. High cell viability was confirmed (Figure S5, Experimental Section), and neurons established contact with the artificial spines, extending their initial processes triggered by the underlying array architecture as shown in the scanning electron micrographs of Figure 1C, suggesting an initial role of artificial spines’ shape in influencing anchoring sites, providing support and stability [48, 57].

**FIGURE 2:**
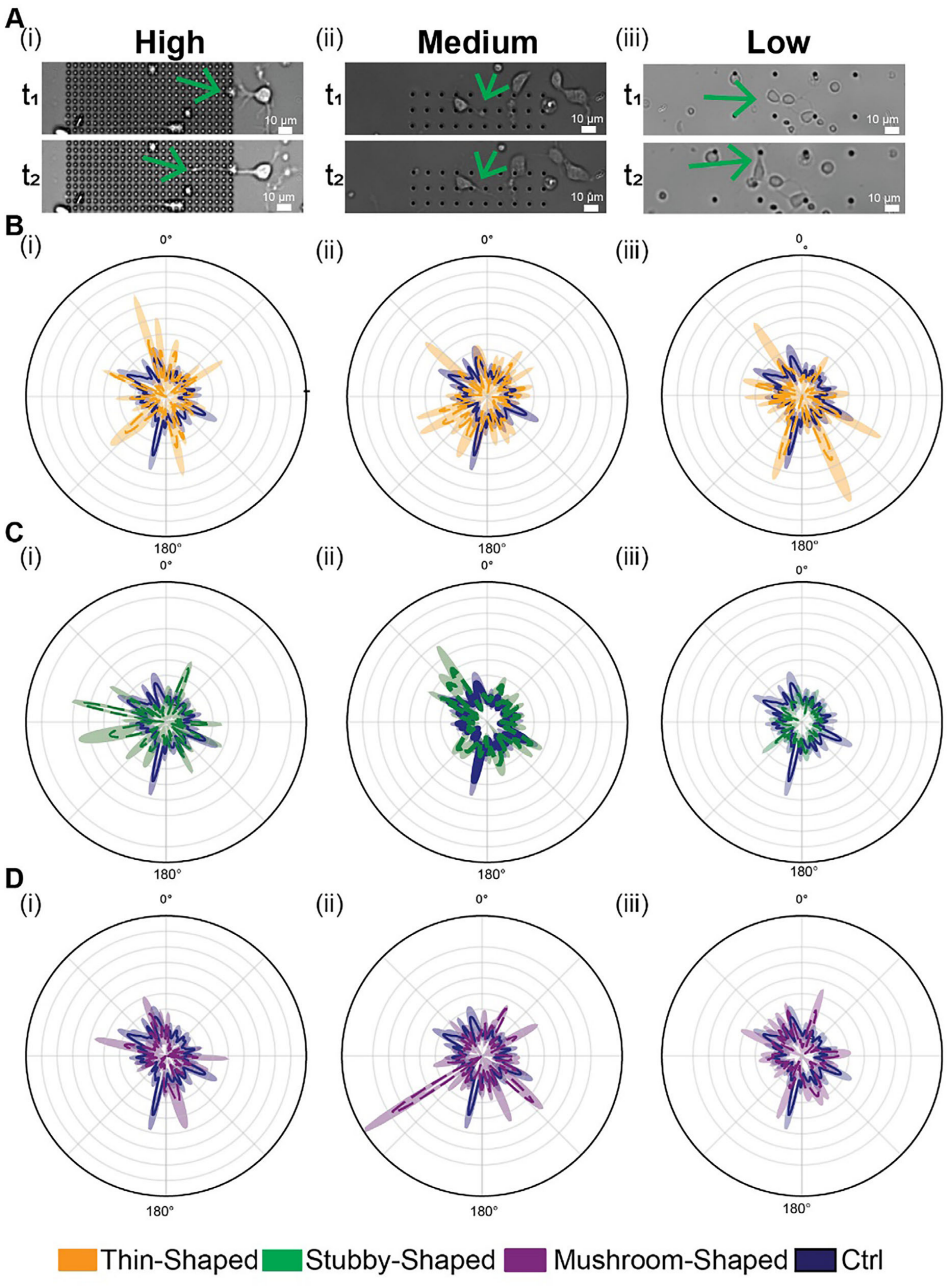
Analysis of neurites outgrowth: (A) Representative phase‐contrast frames selected from time‐lapse video recordings of neurons cultured on surfaces with (i) high, (ii) medium, and (iii) low microstructures’ densities at two time points (t_1_ and t_2_). Green arrows indicate the direction of neurite outgrowth. (B–D) Polar plots representing neurite extension angles over time, measured on (B) thin microstructures (orange, standard deviation shadows) in (i) high, (ii) medium, (iii) low density arrays, (C) stubby microstructures (green, standard deviation shadow) in (i) high, (ii) medium, (iii) low density arrays, and (D) mushroom‐shaped microstructures (magenta, standard deviation shadow) in (i) high, (ii) medium, (iii) low density arrays. Polar plots from control substrates are shown in purple, standard deviation shadow. N = 3 independent experiments were performed, at least 15 neurites analyzed per condition per sample.

We then performed brightfield live imaging over a 24‐h period (Videos S1–S9), identifying regions of interest (ROIs) over time (Figure 2) to investigate how biomimetic microstructures influence initial growth cone (GC) navigation and neurites’ dynamics during this exploring phase [58, 59]. In high‐density microstructure arrays, neuronal interactions induced distinct morphological changes, as shown across live imaging frames (Figures S6–S8; control flat substrate in Figure S9). Initially the GC transitioned from a branched morphology (Figure 2A(i), t_1_) to a bullet‐like shape (Figure 2A(i), t_2_), suggesting a directed movement under spatial confinement (Videos S1–S3). In medium‐density arrays, neurites displayed a sprouting morphology (Figure 2A(ii), t_1_) prior to further extension (Figure 2A(ii), t_2_) (Videos S4–S6). In contrast, low‐density arrays (Figure 2A(iii), t_1_) provided larger planar surface between micropillars, promoting enhanced neurite branching and exploratory growth behavior (Figure 2A(iii), t_2_) (Videos S7–S9).

Neurite extension upon contact with artificial spine arrays (Figure 2A) was analyzed through directionality analysis (Experimental Section) represented in polar plots (Figure 2B–D), where concentrated peak indicates directional preference, with the amplitude reflecting the extent of elongation [57].

In high‐density arrays (Figure 2A(i)), neurites interacting with thin (Figure 2B(i)) and stubby (Figure 2C(i)) artificial spines exhibited reduced angular variability and more centralized growth, suggesting structured guidance. Figure 2D(i) shows that both mushroom‐shaped spines and the control substrate (purple line) display broader orientation distributions compared to other spine types. In medium‐density arrays (Figure 2A(ii)), neurite elongation was more pronounced, particularly for thin (Figure 2B(ii)) and mushroom‐shaped spines (Figure 2D(ii)), with an angular dispersion increase compared to high‐density arrays, especially in the mushroom‐shaped structures. In low‐density arrays (Figure 2A(iii)), neurite extension showed the highest angular distribution across all morphologies, particularly for thin (Figure 2B(iii)) and stubby (Figure 2C(iii)) spines. Interestingly, the mushroom‐shaped (Figure 2D(iii)) structures promoted a more focused directionality, although with increased variability relative to higher‐density structures. The control group (Figure 2D(iii), purple line) also displayed larger angular spread, reflecting reduced spatial confinement.

Across all density conditions, thin spines consistently showed higher angular dispersion compared to the control, with the highest increase occurring in low‐density arrays. These results collectively suggest that spine morphology and array density modulate neurite guidance by shaping spatial constraints and directional variability. In particular, high‐density arrays appear to restrict neurite outgrowth, resulting in reduced angular dispersion, while medium‐density arrays promote greater directional variability and elongation, especially for thin and mushroom‐shaped spines, as shown in Figure 2B(ii),D(ii) [47].

In addition, Figure S10 reports on the average neurite velocity calculated as the difference between the length measured after 24 h and the initial length, divided by the corresponding time interval.

**FIGURE 3:**
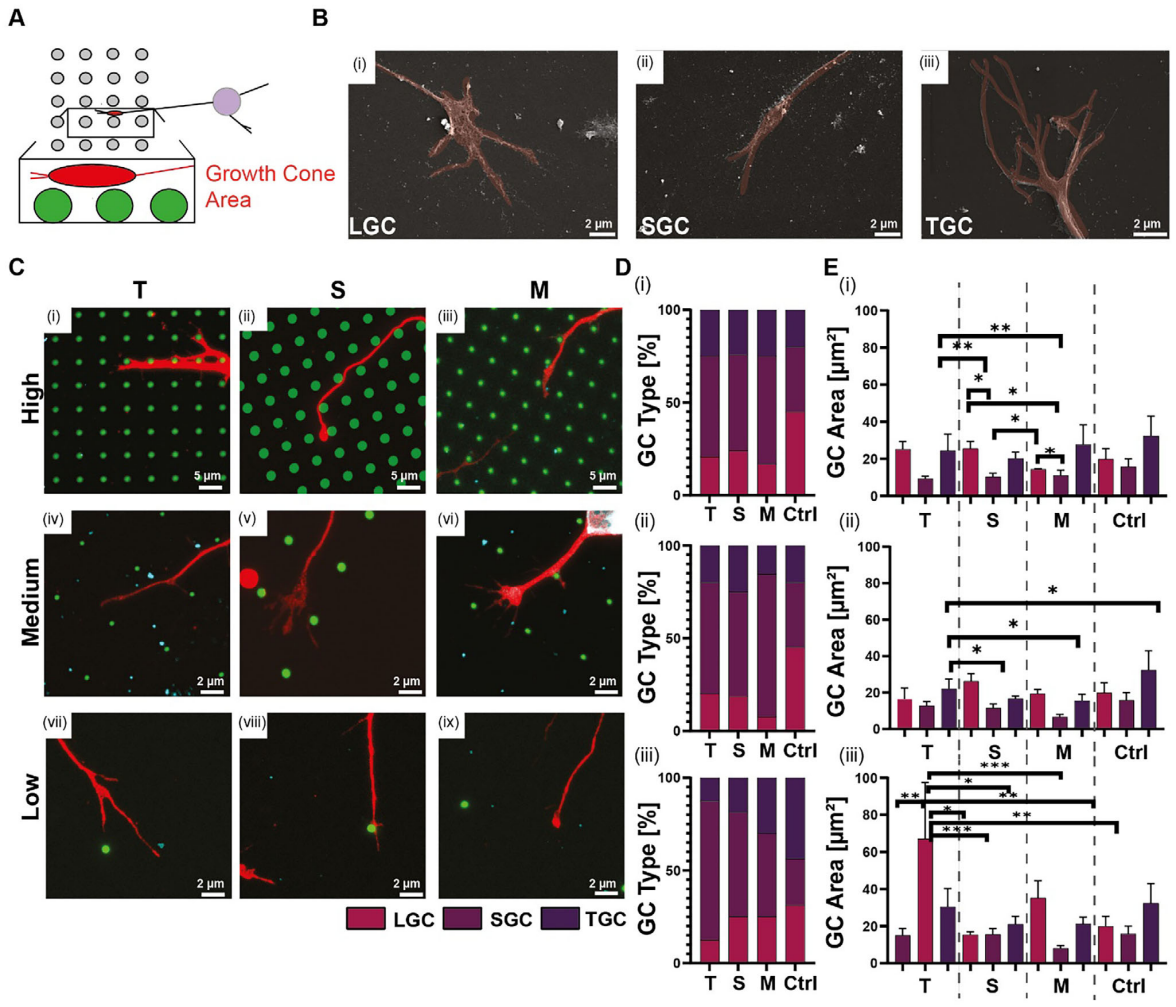
Effect of artificial spines on growth cone morphology. (A) Schematics showing the region of interest considered for the growth cone (GC) morphology analysis. B) Scanning electron micrographs on a flat substrate of (i) large growth cone (LGC), (ii) small growth cone (SGC), and (iii) tree‐like growth cone (TGC). C) Immunostaining of the GC (F‐ actin in cyan‐ 546 nm, β‐III tubulin in red ‐ 647 nm and control in Figure S13) on high‐density arrays (HD), scale bar 5 µm, (i) thin microstructures, (ii) stubby microstructures, (iii) Mushroom‐shaped microstructures; on medium‐density arrays (MD) for (iv) thin microstructures, (v) stubby microstructures, (vi) mushroom‐shaped microstructures and low‐density arrays (LD) for (vii) thin microstructures, (viii) stubby microstructures, (ix) mushroom‐shaped microstructures. (D) Percentage of different growth cone morphology across each substrate (i) high density (HD), (ii) medium‐density (MD), (iii) low density arrays (LD). (E) Quantification of the average area of the GC morphologies, (i) high‐density arrays, (ii) medium‐density arrays, (iii) low‐density arrays (1 DIV). E(i), E(ii), and E(iii) were analyzed using two‐way ANOVA to assess the effects of two independent variables: growth cone morphology and topographical condition. In all panels, the topographical condition had a statistically significant effect (E(i): p = 0.0155; E(ii): p = 0.0268; E(iii): p = 0.0010), whereas the growth cone morphology did not (p > 0.05). Statistically significant differences are indicated in the figures with asterisks (^*^ *p* < 0.05, ^**^ *p* < 0.01, ^***^ *p* < 0.001). N = 3 independent experiments were performed per condition.

To further characterize neurite dynamics, we analyzed the retraction phase by quantifying changes in velocity and length (Figures S11 and S12; Experimental Section). During retraction, neurites exhibited a progressive shortening complemented by a decrease in velocity, suggesting an active reorganization of the cytoskeleton. These observations are consistent with morphological remodeling at the neurite terminal, potentially linked to changes in adhesion or intracellular tension. These observations suggest changes in the morphology of the neurite terminal part, which was further characterized by considering those with the growth cone (axons) and investigating its morphology after 1 day in vitro (DIV, Figure 3A). Here, the growth cone is effectively the neuron's “sensor,” continuously interpolating signals from the surrounding environment, including physical, chemical, and topographical cues. In the context of cell‐material interactions that promote specific neural behaviors, such as axon guidance, the GC plays a crucial role especially in response to specific properties of the material, such as topography [60, 61, 62].

Here, we identified three configurations: a large growth cone (LGC) with a broad area and quasi‐elliptical protrusion (Figure 3B(i)), a small growth cone (SGC) with a bullet shape (Figure 3B(ii)), and a tree‐like growth cone (TGC) with branched axon terminals (Figure 3B(iii)). Each configuration represents a specific neuronal process: LGC corresponds to a pausing state, the wide spread of the cone enables it to collect stimuli from local cues that influences its direction [63]. In the SGC (streamlined growth cone) state, the growth cone becomes more confined, with few and short filopodia, with potential forward movement rather than extensive exploration. This reduction in size reflects increased efficiency, allowing the axon to advance rapidly in response to guidance cues.

In contrast, the TGC enters an exploratory state, extending multiple filopodia and branches in a tree‐like pattern, enabling it to probe multiple directions simultaneously. This morphology suggests that the axon is in a decision‐making phase, actively exploring potential paths in response to a complex environment with competing signals [26, 58, 62, 64, 65], provided here by the artificial spine shape and density.

We characterized the GC area using immunohistochemistry labeling of F‐actin, β‐III tubulin, and the plasma membrane (Experimental Section, control substrate in Figure S13) [26, 50]. High‐density artificial spine arrays predominantly supported small growth cone morphology (Figure 3C(i)–(iii)), while lower‐density arrays (with greater inter‐microstructure spacing) favored larger growth cones (Figure 3C(iv)–(vi)). In low‐density artificial spine arrays, SGC morphology was favored (Figure 3C(vii)–(ix)). Figure 3D summarizes the percentage of growth cones populations: notably, SGC morphology was consistently more prevalent than in controls, regardless of artificial spine shape or density (Figure 3D(i)). In medium‐density arrays, mushroom‐shaped microstructures effectively enhanced SGC formation (Figure 3D(ii)). For low‐density arrays (Figure 3D(iii)), thin microstructures predominantly supported SGC morphology, while stubby shapes also favored SGC, though not as prominently, suggesting that the frequent occurrence of SGC morphology may indicate an active neuronal state.

To explore this further, we analyzed the average growth cone area across different artificial spine arrays (Figure 3E). In particular, LGCs exhibited the largest surface area in high‐density arrays, significantly larger than other configurations (Figure 3E(i)). Interestingly, medium‐density arrays, regardless of microstructures’ shape, induced the bullet‐like SGC morphology (Figure 3E(ii)) more often than in control samples (Figure 3E(iii)). Overall, these results suggest that neuronal growth cone morphology and area respond differentially to artificial dendritic spines depending on both shape and density. At high density, thin‐shaped artificial spines significantly promoted GC expansion [48, 58]. In contrast, at medium and low densities, the influence of mushroom‐shaped microstructures decreased, suggesting that neuronal responsiveness to surface features decreases as spine density decreases [66].

These findings highlight the dynamic responsiveness of neuronal growth cones to variations in surface topography and density during early development. To build on this, we then investigated how neurons interact with artificial dendritic spines at a later developmental stage (4 DIV). Here, neuronal adhesion serves as a key indicator of cell–surface biorecognition, primarily mediated by integrins [21, 38, 43, 44]. Integrins are transmembrane receptors that facilitate interactions between cells and the extracellular matrix. Paxillin, a focal adhesion protein, connects integrins to various structural and signaling components within the cytoplasm [38].

**FIGURE 4:**
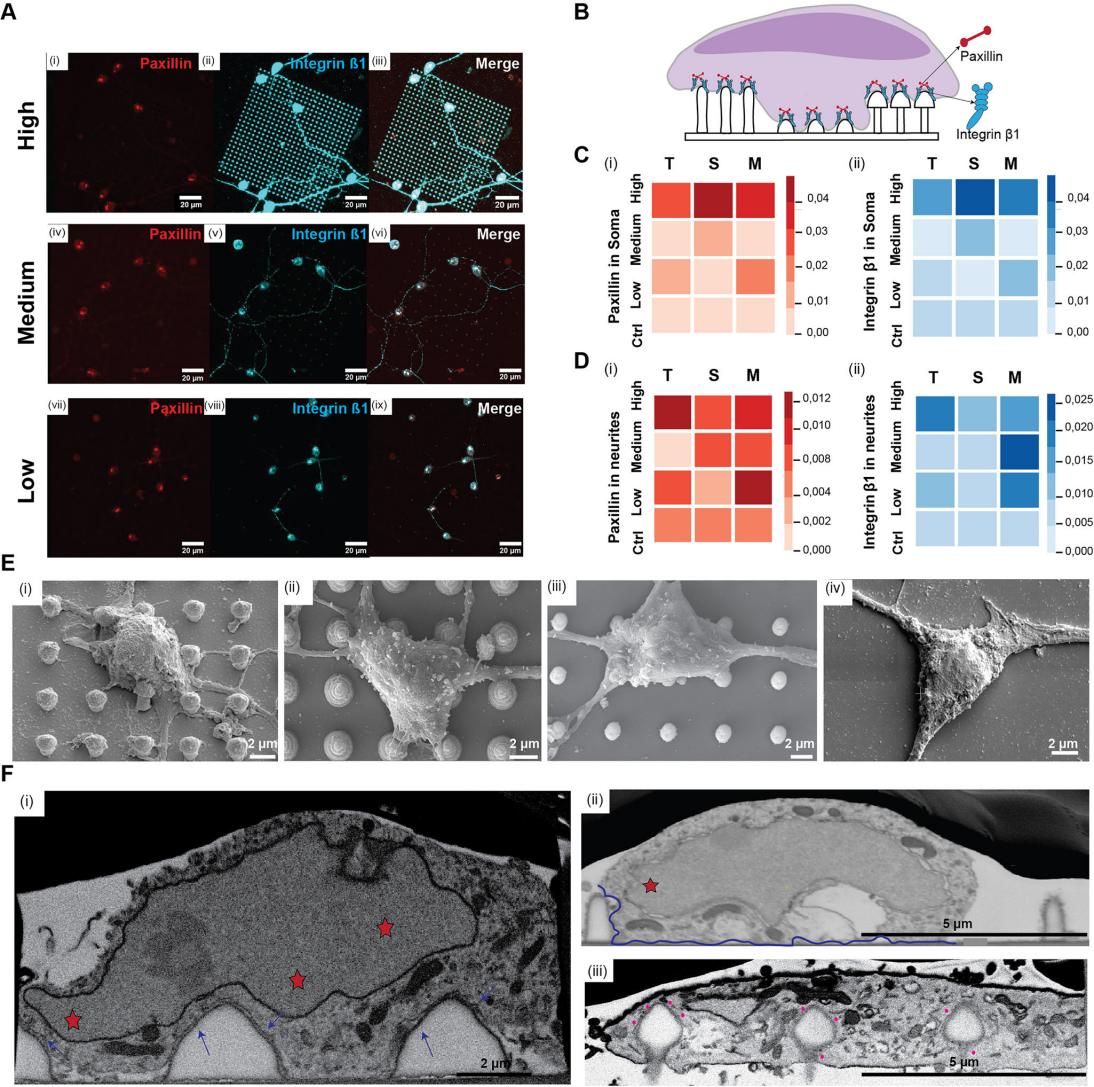
Neuronal adhesion on biomimetic artificial spines. (A) Representative immunofluorescence images showing the distribution of paxillin (red, 647 nm) and integrin β1 (cyan, 546 nm) in cells cultured on substrates with different structures’ densities: (i–iii) high, (iv–vi) medium, and (vii–ix) low. (i, iv, vii) Merged images demonstrate colocalization and cell morphology relative to the substrate patterns. (B) Schematic representation of integrin recruitment and focal adhesion formation. (C) Fluorescence intensity color maps for soma localization of (i) paxillin and (ii) integrin β1 expression; (D) Fluorescence intensity color maps for neurites localization of (i) paxillin, significant effect of treatment group on fluorescence Kruskal‐Wallis test (p = 0.0128) (p = 0.0468). Turkey HSD tests and (ii) integrin β1 expression Kruskal‐Wallis test (p = 0.0219). (E) Scanning electron micrographs of single cells on microstructure arrays (i) mushroom‐ shaped microstructures, (ii) stubby microstructures, (iii) thin microstructures, (iv) flat area. (F) FIB‐milled cross‐sections showing interface and nucleus/membrane deformation on (i) stubby microstructures, (ii) thin microstructures, (iii) mushroom‐shaped microstructures.

In this context, neuronal adhesion was assessed by quantifying the expression of paxillin (Figure 4A(i),(iv),(vii)) and integrin β1 (Figure 4A(ii),(v),(viii)) through immunohistochemistry (Experimental Section, merged micrographs in Figure 4A(iii),(vi),(ix); control substrate in Figure S14). Prior to staining, the artificial spine arrays were treated with Sudan Black B (SBB) to reduce autofluorescence (Experimental Section, Figure S15) [67]. The integrin β1–paxillin complex (Figure 4B) plays a central role in regulating cell adhesion and movement, particularly in response to topographical cues such as vertical micro‐ and nanostructures [68, 69].

Fluorescence intensity maps of proteins labeled at soma (Figure 4C) and neurites (Figure 4D) reveal distinct protein expression patterns (Experimental Section, and segmentation in Figure S16). Within the soma, paxillin expression indicates enhanced recruitment in response to high‐density arrays across all spine shapes (Figure 4C(i)). Similarly, integrin β1 expression is elevated under these conditions (Figure 4C(ii)), particularly in the presence of stubby and mushroom‐shaped microstructures. These results suggest that neuronal cells exhibit a stronger adhesion response to high‐density arrays, with increased recruitment of focal adhesion proteins in both neurites and soma, most prominently in the soma. Here, the paxillin and integrin β1 show a broadly similar trend across conditions, with higher fluorescence observed in the T condition compared to S and M. While their distributions differ in magnitude and organization, this partial correspondence may reflect coordinated, yet distinct, roles in adhesion‐related processes. In neurites, paxillin‐related fluorescence intensity is greater in high‐density arrays across all artificial spines (Figure 4D(i)). In contrast, integrin b1 fluorescence signal displays a stronger sensitivity to substrate architecture than to density alone. Mushroom (M) substrates consistently induce higher integrin expression across medium and low densities, indicating that larger or more complex substrate features favor integrin‐mediated adhesion. Thin (T) substrates also promote integrin expression, particularly at high density, whereas stubby (S) substrates elicit comparatively weaker integrin signals. Control conditions show minimal integrin expression, confirming that integrin upregulation is driven by direct substrate engagement. (Figure 4D‐(ii) [68]

Scanning electron micrographs (Figure 4E) show the morphology of neuronal cells on high density thin (Figure 4E(i)), stubby (Figure 4E(ii)), mushroom‐shaped (Figure 4E(iii)) microstructures and control on flat substrates (Figure 4E(iv)): extensions from the cell bodies appear to spread directionally, likely influenced by the underlying artificial spines, also suggesting topography‐guided growth [48].

Among the shapes tested, mushroom‐shaped artificial spines appear most effective in promoting adhesion, likely due to their larger surface area, greater curvature, and increased potential for membrane wrapping [43, 46]. This wrapping effect, accompanied by local membrane invaginations, was also observed in scanning electron microscopy/focused ion beam (SEM/FIB) cross‐sections (Figure 4F). These images revealed that artificial spines establish close contact with the plasma membrane and induce deformation and adaptation not only of the membrane itself but also of intracellular organelles (Figure 4F(i)–(iii); Figure S17) as also depicted in 3D reconstruction after sequential cross‐sectioning of a volume of interest (Figure S18).

**FIGURE 5:**
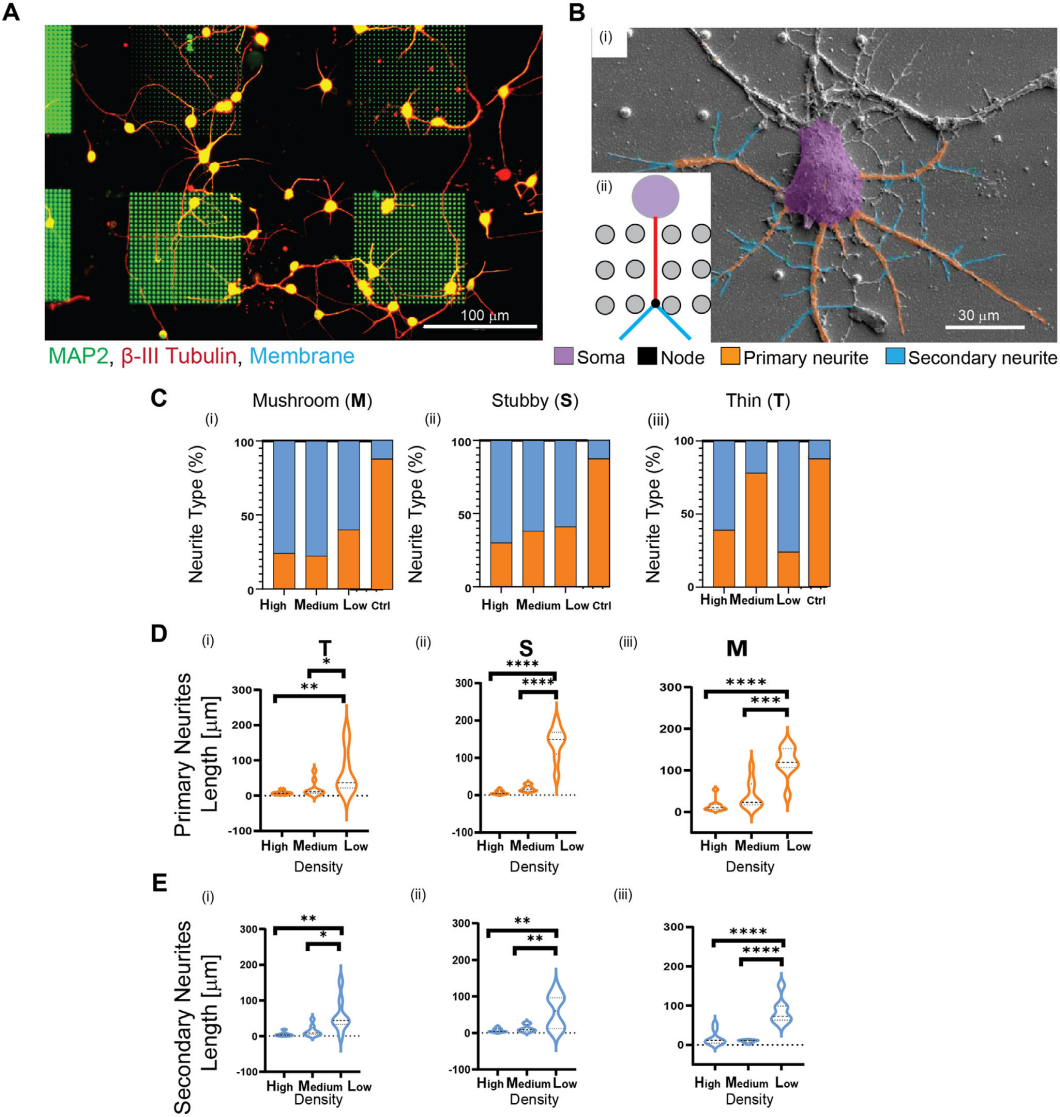
Neuronal network segmentation and analysis. (A) Neuronal morphology visualized using immunofluorescence staining: microtubules and tubulin are marked with β‐III tubulin (red, 647 nm) and MAP2 (green, 488 nm). (B) Schematics representing the segmentation performed for each analyzed neuronal cell: SEM of primary neurons, false‐colored to highlight different neuronal regions of interest. (C) Percentage of primary and secondary neurites normalized to the total cell number: (i) thin microstructures, (ii) stubby microstructures, (iii) mushroom‐shaped microstructures, each for high‐density array (0,28 spines/µm^2^), medium‐density array (0,11 spines/µm^2^) and low‐density array (0045 spines/µm^2^). D(i): ^**^ *p* = 0.0046; ^*^ *p* = 0.0208; D(ii): ^****^ *p* < 0.0001; D(iii) ^****^ *p* < 0.0001, ^***^ *p* = 0.0002). N = 3 independent experiments were performed per condition. D) Statistical analysis of neurite length normalized on number of artificial spines touched: primary neurite length for (i) thin microstructures, (ii) stubby microstructures, (iii) mushroom‐shaped microstructures; (E) secondary neurite length for (iv) thin microstructures, (v) stubby microstructures, (vi) mushroom‐shaped microstructures, each for high‐density array (0,28 spines/µm^2^), medium density array (0,11 spines/µm^2^) and low density array (0045 spines/µm^2^). E(i): ^**^ *p* = 0.0032; ^*^ *p* = 0.0208132; E(ii): ^**^ *p* = 0.0028; E(iii) ^****^ *p* < 0.0001).

Building upon these structural and adhesion‐related observations, we last explored how different artificial spine configurations influence broader aspects of neuronal morphology, specifically neurite outgrowth and branching [70]. Figure 5A presents an exemplary optical micrograph of neuronal cells (cultured on artificial spines labeled against β‐III tubulin, MAP2, and membrane markers (Experimental Section and Figures S19 and S20). Distinct cellular components were identified (Figure 5B(i)), and each neuron was segmented to characterize the cell body (soma), primary neurites (initial projections from the soma), and secondary neurites (branches from primary neurites). Bifurcation points, namely nodes, were also identified (Figure 5B(ii)), and the percentage of primary versus secondary neurites across each substrate (Figure 5C). Among all substrates, medium‐density mushroom‐shaped microstructures had the most significant impact on neurite development, with a higher proportion of secondary neurites observed (Figure 5C(i)). In contrast, stubby microstructures at low density were most effective for overall neurite formation, showing nearly equal proportions of primary and secondary neurites (Figure 5C(ii)). For thin microstructures, medium‐density arrays supported neurite development, though predominantly limited to primary neurites with fewer branches (Figure 5C(iii)). These findings suggest that neurons respond to both the density and shape of artificial spines, with certain topographies preferentially supporting either primary or secondary neurite outgrowth. Moreover, higher densities generally promoted secondary branching, medium densities yielded shape‐dependent responses, and lower densities favored primary neurite formation. Notably, mushroom‐shaped microstructures resulted in reduced neurite elongation, with growth appearing more dispersed across multiple directions [48, 66, 71]. To further assess the influence of artificial spines on neurite elongation, we calculated the average length of primary and secondary neurites, normalized by both the total number of cells, indicating how overall outgrowth varies with spines density and shape. In contrast, neurons cultured on control (flat) substrates exhibited significantly shorter neurites, confirming the role of microstructured surfaces in promoting neurite extension (Figure S22, SEM images in Figure S23, Experimental Section). To specifically evaluate the contribution of contacted microstructures, Figure 5D–E reports neurite length normalized by the number of artificial spines physically touched by each neurite (Experimental Section, Figure 5D,E). Here, the microstructures’ density plays a key role in supporting neurite elongation, likely by providing physical support that prevents collapse. Thin microstructures had minimal effect on neurite length (Figure 5D(i),E(i)), whereas stubby microstructures enhanced neurite extension at low density (Figure 5D(ii),E(ii)). Interestingly, mushroom‐shaped microstructures significantly increased the normalized length of primary neurites (Figure 5D(iii)) and showed a similar behavior for secondary neurites (Figure 5E(iii)) [72]. Together, these results indicate that the density and shape of artificial spines influence not only neurite outgrowth but also directionality (Figures S24 and S25). Microstructures likely act as anchoring points, helping guide neurite extension and stabilize mechanical forces required for growth [73]. Here, the results suggest that both the geometry and density of biomimetic dendritic microstructures strongly influence neurite directionality. At high density, T‐shaped structures induced largely random growth; S‐shaped structures promoted strong alignment, and M‐shaped structures produced moderate alignment. At medium density, all shapes showed increased directionality, with S‐shaped and especially M‐shaped structures exhibiting pronounced alignment along specific angles. At low density, directional guidance decreased overall, particularly for S‐ and M‐shaped microstructures, while T‐shaped structures induced only mild preferential orientation. Overall, these results indicate that artificial dendritic spines serve as topographical cues that guide and stabilize neurite outgrowth, with S‐shaped structures providing the strongest directional guidance, especially at higher densities, whereas T‐ and M‐shaped structures show density‐dependent and more variable effects.

## 3 Conclusions

Our study provides significant insights into the relationship between neuronal cells at early stages and biomimetic microstructures designed to emulate dendritic spines architecture. Through the development and use of neuromorphic microstructures, specifically thin, stubby, and mushroom‐shaped microstructures, this work has explored the influence of these on neuronal outgrowth, growth cone morphology, network development, and cell adhesion. The study demonstrated that microstructure density and shape are critical factors in directing neuronal behavior. High‐density arrays, particularly those with mushroom‐shaped microstructures, were shown to significantly enhance secondary neurite development and guide axon elongation. Thin microstructures, although less influential in altering neurite branching, played a distinct role in modulating the retraction and elongation phases of axon outgrowth. This suggests that specific microstructures can be tailored to influence different aspects of neuronal development, depending on the desired application. Furthermore, the investigation into growth cone morphology revealed that different microstructure configurations can induce distinct neuronal states. High‐density arrays predominantly supported the formation of small, bullet‐shaped growth cones, indicative of an active neuronal state, whereas low‐density arrays favored larger, pausing growth cones. This variation in growth cone morphology, influenced by the spatial arrangement of the microstructures, underscores the importance of topographical cues in neuronal pathfinding and network formation. The study also highlighted the role of microstructures in enhancing cellular adhesion through the engagement of focal adhesion proteins and integrins. SEM and FIB‐SEM analyses provided insights on membrane deformations and cellular engulfment around the microstructures, indicating a strong biorecognition response. These interactions suggest that the designed microstructures not only support neurite outgrowth but also play a pivotal role in stabilizing and guiding neuronal processes and may be involved in influencing the formation and strength of synaptic connections. In conclusion, this research advances the understanding of how biomimetic microstructures can be engineered to influence neuronal behavior. By mimicking the natural environment of neural tissues, these neuromorphic materials hold great potential for applications in neuroengineering, including the development of more effective neural interfaces, prosthetics, and treatments for neurological disorders. The ability to systematically design and optimize these microstructures opens new avenues for the precise control of neuronal growth and network formation, bridging the gap between artificial systems and biological tissues.

## 4 Experimental Section

### 4.1 Fabrication of Artificial Spines Arrays

The microstructures were designed using a CAD software (AutoCAD) and subsequently converted in general writing language (GWL) format with DeScribe software (Nanoscribe GmbH, Germany). The printing parameters, including laser power of 30 mW and scan speed of 10k µm/s, were fixed within the script prepared using the same software (Figure 1; Figures S1–S4 and Table S1).

The fabrication was carried out with Nanoscribe Photonic Professional GT system (Nanoscribe GmbH, Germany), equipped with a 780 nm Ti‐sapphire laser, emitting g ≈ 100 fs pulses at 80 MHz with a maximum power of 150 mW, and a 63 × 1.4 NA oil immersion objective. The microstructures were fabricated employing the commercially available Ip‐Dip resin (Nanoscribe GmbH, Germany), using fused silica as substrate (Nanoscribe GmbH, Germany), in Dip‐in Laser Lithography (DiLL) configuration.

The fabrication process took approx.11 h, followed by development by immersing the sample for 10 min in Mr Dev (5L, micro resist technology, cat. No. A1.08.003‐0001) and subsequently in isopropanol (IPA, Merck Life Science S.r.l., cat. No. 33539‐2.5L‐M) for 10 min, before air drying.

### 4.2 Sterilization and Surface Functionalization of the Substrate

The samples were sterilized under a biological hood by immersion in 70% v/v ethanol (EtOH) (Merck Life Science S.r.l., cat. No. 24105‐2.5L‐M) for 20 min, followed by three washes with autoclaved milliQ water and air drying. Prior to cell plating, the substrate was functionalized by casting a protein solution containing Poly‐L‐lysine (molecular weight 70 000–150 000 Da, Sigma–Aldrich) at 0.01% w/v in water. After incubation, the protein coating was removed, and the substrate was washed with warm Neurobasal medium (Life Technologies, cat. No. 21 103 049).

### 4.3 Decreasing Ip‐Dip Autofluorescence

The Ip‐dip photoresist is an un‐conductive polymer that fluoresces on the green channel. For this reason, The auto‐fluorescence quenching was carried out by immersing the samples in a 0.3% w/v solution of the fluorescence quencher Sudan Black B SBB (Sigma–Aldrich, cat. Num. 2 035 302 986) in 70% ethanol for 2 h. Subsequently, the samples were rinsed in ethanol and they were immersed in phosphate‐buffered saline (PBS Sigma–Aldrich, cat. Num. P4417‐100TAB) for an additional 2 h and then dried using an air gun (Figure S15).

### 4.4 Cell Culture

Primary cortical neurons were obtained from chicken embryo brains, with fertilized chicken eggs (Charles Rivers) incubated for 9 days at 37°C. The brains were dissected and isolated, and cortical hemispheres were extracted and collected in 7 mL of Hibernate medium (Thermo Fisher Scientific, cat. No. A1247501). The tissue was dissociated using 0.25% trypsin‐EDTA (Life Technologies, Italy, cat. No. 25 200 072) for 20 min at 37°C. The resulting suspension was transferred to an Eppendorf tube containing 1 mL of Neurobasal medium (Life Technologies, cat. No. 21 103 049), supplemented with 1% B‐27 (Life Technologies, cat. No. 17 504 044), 0.2% l‐glutamine (Merk Life Science S.r.l., Italy), and 0.2% penicillin‐streptomycin (Sigma–Aldrich, cat. No. P4333‐100ML). After manual centrifugation, the tissue was mechanically triturated with pipettes of varying sizes (1000, 200, and 20 µL), ensuring that droplet formation was avoided. The number of viable cells was assessed using trypan blue 0.4% (Invitrogen, Thermo Fisher Scientific, USA) and counted automatically with a Countless II Automated Cell Counter (Thermo Fisher Scientific, cat. No. C10228). The desired cell density (42k cells/cm^2^) was then plated on Poly‐L‐lysine coated substrates.

### 4.5 Viability Assay

Biocompatibility was assessed after 1 day in vitro (DIV) using Calcein acetoxymethyl ester (Calcein‐AM, Merck Life Science S.r.l., Italy) with a 1:1000 dilution in phosphate‐buffered saline (PBS) (Sigma–Aldrich, cat. Num. P4417‐100TAB) to label live cells. Additionally, 1 µg/mL of Ethidium homodimer (Sigma–Aldrich, cat. No. 46 043) was added and incubated for 15 min at 37°C. Samples were then washed with warm PBS prior to imaging (Figure S5).

The number of live and dead cells was quantified using Image‐J software, and cell viability was calculated as follows:

$$\mathrm{viability}=\left(\mathrm{Live}\;\mathrm{cells}/\left(\mathrm{live}\;\mathrm{cells}+\mathrm{dead}\;\mathrm{cells}\right)\right)\cdot 100$$

### 4.6 Immunohistochemistry

#### 4.6.1 Growth Cone Labeling

Samples were fixed at 1 DIV using 4% v/v paraformaldehyde (PFA) (Società Italiana Chimici, cat. No. 15 710) in PBS (Sigma–Aldrich, cat. No. P4417‐100TAB) for 20 min, followed by rinsing three times in warm PBS. The samples were then immersed in 0.1% v/v Triton X‐100 (Merck, cat. No. X100‐100ML) in PBS for 5 min and then washed three times with PBS. Specimens were blocked in 2% w/v Bovine Serum Albumin (BSA) (Sigma–Aldrich, cat. No. A2153‐100G) in PBS for 45 min at room temperature (RT) and then washed three times with PBS. Neuronal cells were labeled with Phalloidin‐iFluor 555 Reagent conjugated dyes (1:1000 dilution in PBS, Abcam, cat. No. cat. num. ab176756), for 1 h at room temperature (RT), following three washing with 2% w/v BSA. Alexa Fluor 546 (dilution 1:500 for 30 min, anti‐mouse, λEX/EM = 490/525 nm) (Thermo Fisher Scientific cat. No A11030). Ab II for anti‐β‐III tubulin (Abcam cat. No. Ab78078) was diluted (1:1000 dilution, in 2% BSA) and let incubate for 1 h at RT. Cells were stained with CellMask Deep Red plasma membrane stain (Invitrogen, Thermo Fisher Scientific, Cat. No. C10046) at a dilution of 1:1000 in culture medium. Staining was performed at room temperature for 15 min in the dark. Images were acquired using a 63x objective lens, and Image‐J software was used to quantify the growth cone area (Figure 3; Figure S13).

#### 4.6.2 Focal Adhesion Proteins, Integrin Labeling

After 4 DIV, cells were fixed using 4% v/v paraformaldehyde (PFA) (Società Italiana Chimici, cat. No. 15 710) in PBS (Sigma–Aldrich, cat. No. P4417‐100TAB) for 20 min, followed by rinsing three times in warm PBS. The samples were then immersed in 0.1% v/v Triton X‐100 (Merck, cat. No. X100‐100ML) in PBS for 5 min and then washed three times with PBS. Specimens were blocked in 2% w/v Bovine Serum Albumin (BSA) (Sigma–Aldrich, cat. No. A2153‐100G) in PBS for 45 min at room temperature (RT) and then washed three times with PBS. Paxillin (Life Technologies, cat. No. AHO0492) was labeled with a monoclonal antibody (mouse), diluted in BSA (1:200) for 1 h, followed by labeling with anti‐mouse Alexa Fluor 546 (1:500 for 30 min). Integrin β1 was labeled with a phosphor T788 + T789 antibody (ab5189), and then with anti‐rabbit Alexa Fluor 647 (Thermo Fisher Scientific, cat. No. A21443, 1:500 for 30 min) (Figure 4; Figure S14).

#### 4.6.3 Cytoskeleton, Neurites, and Nuclei Labeling

(Figure 5A; Figures S19 and S20). Samples were fixed at 4 DIV using 4% v/v paraformaldehyde (PFA) (Società Italiana Chimici, cat. No. 15 710) in PBS (Sigma–Aldrich, cat. No. P4417‐100TAB) for 20 min, followed by rinsing three times in warm PBS. The samples were then immersed in 0.1% v/v Triton X‐100 (Merck, cat. No. X100‐100ML) in PBS for 5 min and then washed three times with PBS. Specimens were blocked in 2% w/v Bovine Serum Albumin (BSA) (Sigma–Aldrich, cat. No. A2153‐100G) in PBS for 45 min at room temperature (RT) and then washed three times with PBS. Neurons were labeled with anti‐β‐III tubulin (mouse, Abcam AB78078) and anti‐MAP2 (rabbit, SYSY 18 800) antibodies. Samples were incubated with anti‐MAP2 Alexa Fluor 488 (rabbit, 1:500) and anti‐tubulin Ab III Alexa Fluor 546 (mouse, 1:500) for 30 min, followed by staining with the nuclear marker Hoechst, and then they were stained with CellMask Deep Red plasma membrane stain (Molecular Probes, Invitrogen, Thermo Fisher Scientific, Cat. No. C10046) at a 1:1000 dilution in culture medium. The staining was performed at room temperature for 15 min in the dark. acquired using a 63x objective lens.

### 4.7 Optical Imaging

#### 4.7.1 Live Imaging

Live imaging was performed using a microscope equipped with an incubator support, with controlled humidity and temperature (37°C, dark) for the first 24 h post‐seeding. Image acquisition was performed in brightfield condition, using an inverted fluorescence motorized microscope (Axiobserver‐Z1, Zeiss, Germany) (Figure 2; Figures S6–S9 and Videos S1–S9).

#### 4.7.2 Viability Assay

Images were acquired using a widefield fluorescence microscope (Axiobserver‐Z1, Zeiss, Germany) operated through epifluorescence and equipped with a 20x/ 0.8 numerical aperture (NA) water immersion objective lens (Figure S5).

#### 4.7.3 Immunohistochemistry

All samples were prepared with a 50 µL glycerol solution (30% v/v, Sigma–Aldrich, cat. No. G5516‐100ML) and sealed with glass coverslip. Imaging was performed using laser scanning microscope (STED‐SP5, Leica) using a 63x/1.4 NA oil immersion objective, and 100x/1.4 NA oil immersion objective. Images were acquired fixing a slice thickness of 0.25 µm.

### 4.8 Image Processing and Analysis

#### 4.8.1 Viability Assay

The viability was determined by acquiring 20 frames per experiment from 3 collected over the microstructure's substrates. Each experimental condition was studied in triplicate (N = 3, independent experiments). The acquired images were processed by ImageJ software on each frame derived by the Calcein AM and Ethidium Homodimer labeling. The viability was determined using the following formula (Figure S5):

$$\%\;\mathrm{Viability}\;=\left(\mathrm{Live}\;\mathrm{cells}\;/\;\left(\mathrm{Live}\;\mathrm{cells}+\;\mathrm{Dead}\;\mathrm{cells}\right)\right)\;\times \;100$$

Means values of the amount of live and dead cells were calculated by averaging among all experiments [26].

#### 4.8.2 Analysis of Neurites Outgrowth

Analysis codes can be found here: https://github.com/CHARLESProtocol2/Neurite‐Growth‐Analysis‐Toolkit.git

In brief, manually traced neurites from individual time‐lapse frames were used as the input for all analyses. Tracings were generated in ImageJ as *.ndf* files, each describing a neurite as an ordered series of 2D coordinates. These files were exported to plain‐text format and processed with a custom Python parser that reconstructed the tracing structure, extracted the corresponding frame index from each filename, and assembled all annotations into a unified dictionary containing ordered coordinate paths in pixel units. This dataset formed the basis for geometric and dynamic feature extraction (Figure 2; Figures S6–S12).

For each neurite tracing, core morphological features, including centroid position and total path length, were computed directly from the coordinate sets after applying the appropriate pixel‐to‐micrometer conversion factor. All measurements and associated metadata were consolidated into a single feature‐level DataFrame.

Because neurites were annotated independently at each time point, identities were reconstructed computationally. Each tracing was encoded as a compact feature vector containing centroid coordinates, frame index, length, orientation, and spatial region assignment (pillar or control). A custom distance function integrating spatial, temporal, and morphological differences was used to quantify pairwise similarity among tracings. Neurites that were spatially proximate, occurred in adjacent frames, and shared similar shapes and orientations were considered candidates for representing the same physical neurite over time. The resulting distance matrix was supplied to a DBSCAN‐based clustering procedure, which grouped mutually consistent tracings into trajectories while designating spurious or discontinuous annotations as noise. This approach recovered neurite identities even when structures briefly exited and re‐entered the field of view.

Spatial region labels were assigned by comparing neurite centroids to a binary mask of the micropillar layout. For experiments involving heterogeneous pillar geometries, the field of view was subdivided into vertical segments to differentiate microstructure subtypes. These spatial labels were retained for all downstream quantitative analyses.

Dynamic parameters were computed after trajectory reconstruction. Frame‐to‐frame changes in length were used to identify growth and retraction events. Centroid displacements were calculated in micrometers using the imaging conversion factor, and instantaneous velocities were derived using the known temporal spacing between frames. Distributional inspection across the full dataset showed median displacements of ∼7 µm and median velocities of ∼0.15 µm/min, consistent with reported ranges. Extremely large displacements or velocities, typically reflecting tracking discontinuities, were removed using conservative upper thresholds (≤150 µm displacement per interval; ≤1 µm/min velocity). For each displacement vector, direction was extracted using the arctangent function and used to classify forward or backward motion. All dynamic quantities were compiled into a dedicated movement‐dynamics DataFrame for statistical analysis.

Trajectory integrity was verified visually by overlaying reconstructed paths onto raw microscopy frames. Cluster continuity was evaluated to ensure that trajectories exhibited biologically plausible motion patterns, and outlier clusters were discarded. Final velocity distributions were cross‐checked against empirical expectations to confirm realistic motion magnitude and variability.

Directional analyses (Figure 2) were performed using circular statistics. Movement directions, stored in radians (–π to π), were binned into 36 angular sectors (10° resolution). For each bin, the relative frequency was computed as the percentage of total movements within that angular range. Polar plots were generated with a fixed radial scale (0–10%) to enable consistent comparison across conditions. Each plot included a filled frequency polygon, directional reference lines at principal angles, and summary metadata (sample size and total movement count). These representations facilitated the detection of directional biases and enabled the direct comparison of angular distributions across substrate types.

All analysis scripts were written in Python 3.11 using standard scientific libraries (NumPy, pandas, scikit‐learn, OpenCV, matplotlib) and organized into a modular, class‐based architecture. All results, including summary statistics, processed trajectories, and visualizations, were exported as *.csv* and image files to support reproducibility.

#### 4.8.3 Effect of Artificial Spines on Growth Cone Morphology

The effect of artificial spines on growth cone morphology was investigated acquiring 30 frames per experiment (N = 3 independent experiments), studied in triplicate (n = 3, number of samples per condition). The acquired images were then processed by using ImageJ software and by manually tracing the area (Figure 3).

The growth cone area (Figure 3) was evaluated by using the following formula:

$$\begin{array}{ccc} \%\;\mathrm{LGC}\;\mathrm{morphology} & = & \left(\mathrm{total}\;\mathrm{LGC}\;/\;\mathrm{total}\;\mathrm{GC}\;\mathrm{type}\right)\;\times \;100\\ \%\;\mathrm{SGC}\;\mathrm{morphology} & = & \left(\mathrm{total}\;\mathrm{SGC}\;/\;\mathrm{total}\;\mathrm{GC}\;\mathrm{type}\right)\;\times \;100\\ \%\;\mathrm{TGC}\;\mathrm{morphology} & = & \left(\mathrm{total}\;\mathrm{TGC}\;/\;\mathrm{total}\;\mathrm{GC}\;\mathrm{type}\right)\;\times \;100\\ \mathrm{GC}\;\mathrm{area} & = & \left(\mathrm{total}\;\mathrm{LGC}\;\mathrm{area}\;/\;\mathrm{total}\;\mathrm{cell}\right)\;\times \;100\\ \mathrm{GC}\;\mathrm{area} & = & \left(\mathrm{total}\;\mathrm{SGC}\;\mathrm{area}\;/\;\mathrm{total}\;\mathrm{cell}\right)\;\times \;100\\ \mathrm{GC}\;\mathrm{area} & = & \left(\mathrm{total}\;\mathrm{TGC}\;\mathrm{area}\;/\;\mathrm{total}\;\mathrm{cell}\right)\;\times \;100. \end{array}$$

#### 4.8.4 Cell Adhesion

The methodology involved segmenting and identifying the entire neuronal body in two stages: initially isolating the soma and subsequently segmenting the neurites. The heatmaps were obtained by segmentating using Grabcut and Watershed. Then, the images were used to train a U‐Net model. Indeed, the segmented images were analyzed using a pre‐trained U‐Net model, which calculates the normalized fluorescence intensity as follows [29] (Figure 4):

$$\begin{array}{ccc} & & \mathrm{Fluorescence}\;\mathrm{intensity}\\ & & \;=\left(\mathrm{Cell}\;\mathrm{fluorescence}\;-\;\mathrm{Background}\;\mathrm{fluorescence}\right)/\;\mathrm{Cell}\;\mathrm{area} \end{array}$$

Analysis codes can be found here: https://github.com/EstherMatamoros/ProteinExpression.git

#### 4.8.5 Network Development

The development of the neural network was evaluated to acquire 40 frames per experiment from N = 3 independent experiments, with n = 3 samples per condition per experiment. The acquired images were processed by using NeuronJ. It was possible to trace manually the neurites by quantifying the length, branching and morphology of neuronal structures.

The development of the neural network was evaluated by using the following formulas (Figure 5; Figures S19–S22) [70]:

• Normalized number of nodes = (Total neurites/Total nodes), (Figure S21).
• % Primary neurites = (Primary neurites/Total neurites) × 100 (Figure 5C).
• % Secondary neurites = (Secondary neurites/Total neurites) × 100 (Figure 5C).
• Elongation of primary neurites on microstructures = (Primary neurites length/microstructures touched), (Figure 5D).
• Elongation of secondary neurites on microstructures = (Secondary neurites length/microstructures touched) (Figure 5E).
• Elongation of primary neurites on cells = (primary neurites length/total cell number), (Figure S22A).
• Elongation of secondary neurites on cells = (secondary neurites length/total cell number), (Figure S22B).

#### 4.8.6 Cell Guidance

The cell guidance was evaluated to acquire 40 frames per experiment (N = 3 independent experiments), studied in triplicate (n = 3, number of samples per condition). The acquired images were processed by using NeuronJ plugin and by manually tracing the neurites by highlighting the neurites grow and extending. The saved tracings were then processed by using Origin Pro to obtain a polar plots to visually present the distribution of angles (0°–180°) (Figures S24 and S25).

### 4.9 Statistical Analysis

All experiments were conducted in biological triplicates (N = 3 independent experiments) with samples triplicates (n = 3).

All experiments were performed using three independent biological replicates, corresponding to neuronal cultures obtained from three different tissue samples (chicken embryos). For each biological replicate, cells were seeded and analyzed across six technical replicates per experimental condition, including appropriate controls.

For morphological and neurite outgrowth analyses, data were collected from a total of approximately 15–20 neurons per condition, pooled from the three biological replicates (with equal contribution from each replicate). Neurons were selected for analysis based on clear visibility of soma and neuritic processes, absence of overlapping cells, and appropriate positioning on the microstructured or control substrates.

All quantifications were performed blind to experimental condition, using standardized image processing and measurement protocols.

Statistical analyses were performed using GraphPad Prism 8, with data plotted as the standard error of the mean (SEM). The two‐way ANOVA followed by Tukey's multiple comparisons test was used to evaluate the effect of microstructures on cell viability, growth cone morphology, and neural network development. (Figures 3E and 5D,E)

#### 4.9.1 Scanning Electron Microscopy and Focused Ion Beam Sectioning (SEM‐FIB)

##### 4.9.1.1 Samples Preparation with Critical Point Drying (CPD)

The specimens were chemically fixed in 4% v/v PFA, (Società Italiana Chimici) for 20 min and then rinsed three times in milli‐Q water. The fixation was stabilized by overnight immersion in 2.5% v/v glutaraldehyde (Società Italiana Chimici, cat. No. 16 220) diluted in 0.1 m cacodylate buffer (Società Italiana Chimici, cat. Num 11 655) at 4°C. After rinsing with buffer solution, the samples were incubated in a 20 mm glycine solution (Sigma–Aldrich, cat. No. T6522‐100MG) for 20 min. Post‐fixation was carried out using a 4% v/v aqueous osmium tetroxide solution and 2% potassium ferrocyanide (Electron Microscopy Science) for 1 h at 4°C, in darkness. Dehydration was then performed via a graded ethanol series (30% v/v, 50% v/v, 75% v/v, 2×95% v/v, and 100% v/v ethanol in water), each step lasting 10 min at 4°C. The samples were finally processed in a critical point dryer, where ethanol was gradually replaced with CO_2_. Once dried, the specimens were mounted on aluminum stubs and sputter‐coated with a 15 nm thick layer of gold before imaging. This procedure was used to visualize the growth cone morphology, and to obtain micrographs of neuronal morphologies on microstructure's arrays (Figure S23).

##### 4.9.1.2 Samples Preparation with Ultra‐Thin Plasticization (UTP)

The samples were prepared using an ultra‐thin plasticization procedure. Initially, the biological samples were chemically fixed with 4% v/v PFA, Società Italiana Chimici) in milli‐Q water for 20 min. Following this, the samples were incubated overnight at 4°C in 2.5% v/v glutaraldehyde (Electron Microscopy Science) diluted in 0.1 m cacodylate buffer. During these steps, the samples were kept on ice and rinsed with 0.1 m cacodylate buffer solution. Next, the buffer was replaced with a 20 mm glycine solution in 0.1 m sodium cacodylate, and the samples were incubated for 20 min. The specimens were then placed in a solution containing 4% v/v aqueous osmium tetroxide (Electron Microscopy Science) and 2% potassium ferrocyanide (Electron Microscopy Science) for 1 h at 4°C, protected from light. Subsequently, the samples were washed three times with 0.1 m cacodylate buffer and incubated in 2% v/v aqueous osmium tetroxide solution (Electron Microscopy Science) at room temperature for 30 min. After thorough rinsing with room temperature deionized (DI) water, the specimens were immersed in a 1% filtered thiocarbohydrazide (TCH, Electron Microscopy Science) solution in DI water for 20 min at room temperature, followed by three rounds of rinsing with DI water. The samples were then immersed overnight at 4°C in a 4% v/v uranyl acetate solution (SIC, cat. No. 22400‐4). Following three additional rinses with DI water, the specimens were incubated in a 0.15% v/v tannic acid solution (Sigma–Aldrich) for 3 min at 4°C. Dehydration was performed through a graded ethanol series (30% v/v, 50% v/v, 75% v/v, 2×95% v/v, and 100% v/v ethanol in water), with each step lasting 10 min at 4°C. Additionally, the samples were rinsed twice with 100% ethanol at room temperature [55, 56].

For embedding, the samples were gradually infiltrated with resin (comprising 25 mL NSA, 8 mL D.E.R. 736, 10 mL ERL 4221, and 301 µL DMAE, all from Electron Microscopy Science, Cat. No. 1 863 616) using increasing ethanol‐to‐resin ratios. The initial embedding used a 1:3 ethanol‐to‐resin ratio for 2 h, followed by a 1:2 ratio for another 2 h, then a 1:1 ratio overnight. The final embedding was performed with a 2:1 resin‐to‐ethanol ratio for 2 h, followed by pure resin overnight and throughout the day.

The specimens were then positioned vertically for 3 h and polymerized at 70°C for 36 h. To prepare the samples for imaging, they were mounted on 3.2 mm aluminum stubs using silver conductive paste (RS Pro) and coated with a 15 nm thick layer of gold. This protocol has been used to obtain the cross section of the neurons on microstructures.

##### 4.9.1.3 Scanning Electron Microscopy (SEM)

The investigation of growth cone morphology and the influence of topographical cues on neural behavior, particularly highlighting anchoring, was conducted by using SEM. Additionally, SEM was used to capture micrographs of the microstructures. SEM imaging was performed using a ULPTRAPLUSS ZEISS field emission gun (FEG) SEM. Samples processed with CPD and UTP were imaged at acceleration voltages ranging from 5 to 20 kV, with a working distance of 8–30 mm. Prior to imaging, the samples were coated with a conductive gold layer (15–20 nm thick).

##### 4.9.1.4 Cross Sectioning and Imaging

The cross section of the neurons on microstructures were obtained by using SEM‐FIB. The specimens (processed by UTP procedure) were loaded into the dual‐beam vacuum chamber (Thermo Fisher, Helios CX5). The region of interest (ROI) was identified, and the stage was tilted to 52°. A deposition layer of platinum (0.7 µm thickness, current 0.43 nA, voltage 30 kV) was applied via ion beam deposition. Cross‐sections were created by trenching with the ion beam (milling thickness: 5 µm, current: 0.79 nA, voltage: 30 kV), followed by polishing the interface with the ion beam (current: 0.23–0.43 nA, voltage: 30 kV). Scanning electron micrographs were acquired in backscattered mode at high resolution with a dwell time of 20 µs and an electron beam set to 3 kV and 0.17 nA (Figure 4; Figures S17 and S18).

##### 4.9.1.5 3D Reconstruction

For 3D reconstruction, approximately 300 slices per cell, each with a thickness of 5 nm, were collected using the Slice and View tool, a feature of the FIB‐SEM software that enables automatic capture of successive cross‐sections. Segmentation was performed using Imod software, where each slice was manually or semi‐automatically outlined to differentiate cellular structures from the background. Here, the substrates were purple colored, the nucleus were green, and the cell membrane light blue colored. This process involved assigning distinct labels to specific regions of interest, such as membranes or organelles, across all slices. After segmentation, the labeled regions were aligned and reconstructed in 3D, resulting in a detailed model of the entire cell.

## Author Contributions

C.L.B. contributed to experimental design and execution, data acquisition, analysis, interpretation, and manuscript writing. E.M. contributed to data analysis and manuscript writing. V.M. contributed to experimental execution and manuscript writing. A.M. contributed to experimental execution and manuscript writing. V.C. contributed to experimental design and manuscript writing. F.S. contributed to conceiving, experimental design, data interpretation, supervision and manuscript writing.

## Conflicts of Interest

The authors declare no conflicts of interest.

## Supporting information




**Supporting File 1**: advs73965‐sup‐0001‐SuppMat.docx

**Supporting File 2**: advs73965‐sup‐0002‐VideoS1.avi

**Supporting File 3**: advs73965‐sup‐0003‐VideoS2.avi

**Supporting File 4**: advs73965‐sup‐0004‐VideoS3.avi

**Supporting File 5**: advs73965‐sup‐0005‐VideoS4.avi

**Supporting File 6**: advs73965‐sup‐0006‐VideoS5.avi

**Supporting File 7**: advs73965‐sup‐0007‐VideoS6.avi

**Supporting File 8**: advs73965‐sup‐0008‐VideoS7.avi

**Supporting File 9**: advs73965‐sup‐0009‐VideoS8.avi

**Supporting File 10**: advs73965‐sup‐0010‐VideoS9.avi

## Data Availability

The data that support the findings of this study are available from the corresponding author upon reasonable request.
