# Supplementary material for: How Neuromorphic Microstructures Control In Vitro Early‐Stage Neuronal Outgrowth
Source: Adv Sci (Weinh). 2026 May 19;13(33):e10822. doi: 10.1002/advs.202510822 (PMC13271619; doi:10.1002/advs.202510822)
Supplement: Supplementary file 1 — Supporting File 1: advs73965‐sup‐0001‐SuppMat.docx [file ADVS-13-e10822-s004.docx]

**How neuromorphic microstructures control *in vitro* early-stage neuronal outgrowth**

Claudia Latte Bovio^a,b±^, Esther Matamoros^c,d^, Valentina Mollo^a^, Anna Mariano^a¥^, Valeria Criscuolo^c,d^, Francesca Santoro^a,c,d*^

^a^Tissue Electronics, Istituto Italiano di Tecnologia, 80125 Naples, Italy.   
^b^Dipartimento di Chimica, Materiali e Produzione Industriale, Università di Napoli Federico II, 80125, Naples, Italy  
^c^Neuroelectronic Interfaces, Faculty of Electrical Engineering and IT, RWTH Aachen, 52074, Germany.   
^d^Institute for Biological Information Processing-Bioelectronics (IBI-3), Forschungszentrum Juelich, 52428, Germany.

^¥^current address: Institute of Polymers, Composites and Biomaterials (IPCB) − CNR, Viale J. F. Kennedy 54, Mostra D'Oltremare Pad 20, Naples, 80125 Italy

^±^current address: Center for Biomolecular Nanotechnologies, Istituto Italiano di Tecnologia, Arnesano, 73010 Lecce, Italy

Email: [Claudia.lattebovio@iit.it](mailto:Claudia.lattebovio@iit.it), [matamoros@nei.rwth-aachen.de](mailto:e.matamoros@fz-juelich.de), [valentina.mollo@iit.it](mailto:valentina.mollo@iit.it), annamariano@cnr.it , [criscuolo@nei.rwth-aachen.de](mailto:criscuolo@nei.rwth-aachen.de)

**SUPPLEMENTARY INFORMATION**

**Supplementary Video S1**

**Live imaging of primary neurons on high-density thin microstructures array**

This video shows time-lapse live imaging of primary chicken neurons during the first 24 hours of culture on a high-density, thin microstructures array.

**Supplementary Video S2**

**Live imaging of primary neurons on high-density stubby microstructures array**

This video shows time-lapse live imaging of primary neurons during the first 24 hours of culture on a high-density, stubby microstructures array.

**Supplementary Video S3**

**Live imaging of primary neurons on high-density mushroom-shaped microstructures array**

This video shows time-lapse live imaging of primary neurons during the first 24 hours of culture on a high-density, mushroom-shape microstructures array.

**Supplementary Video S4**

**Live imaging of primary neurons on medium-density thin microstructures array**

This video shows time-lapse live imaging of primary neurons during the first 24 hours of culture on a medium -density, thin microstructures array.

**Supplementary Video S5**

**Live imaging of primary neurons on medium-density stubby microstructures array**

This video shows time-lapse live imaging of primary neurons during the first 24 hours of culture on a medium -density, stubby microstructures array.

**Supplementary Video S6**

**Live imaging of primary neurons on medium-density mushroom-shaped microstructures array**

This video shows time-lapse live imaging of primary neurons during the first 24 hours of culture on a medium-density, mushroom-shape microstructures array.

**Supplementary Video S7**

**Live imaging of primary neurons on medium-density thin microstructures array**

This video shows time-lapse live imaging of primary neurons during the first 24 hours of culture on a low -density, thin microstructures array.

**Supplementary Video S8**

**Live imaging of primary neurons on medium-density stubby microstructures array**

This video shows time-lapse live imaging of primary neurons during the first 24 hours of culture on a low -density, stubby microstructures array.

**Supplementary Video S9**

**Live imaging of primary neurons on medium-density mushroom-shaped microstructures array**

This video shows time-lapse live imaging of primary neurons during the first 24 hours of culture on a low -density, mushroom-shaped microstructures array.

**Supplementary Figure S1**


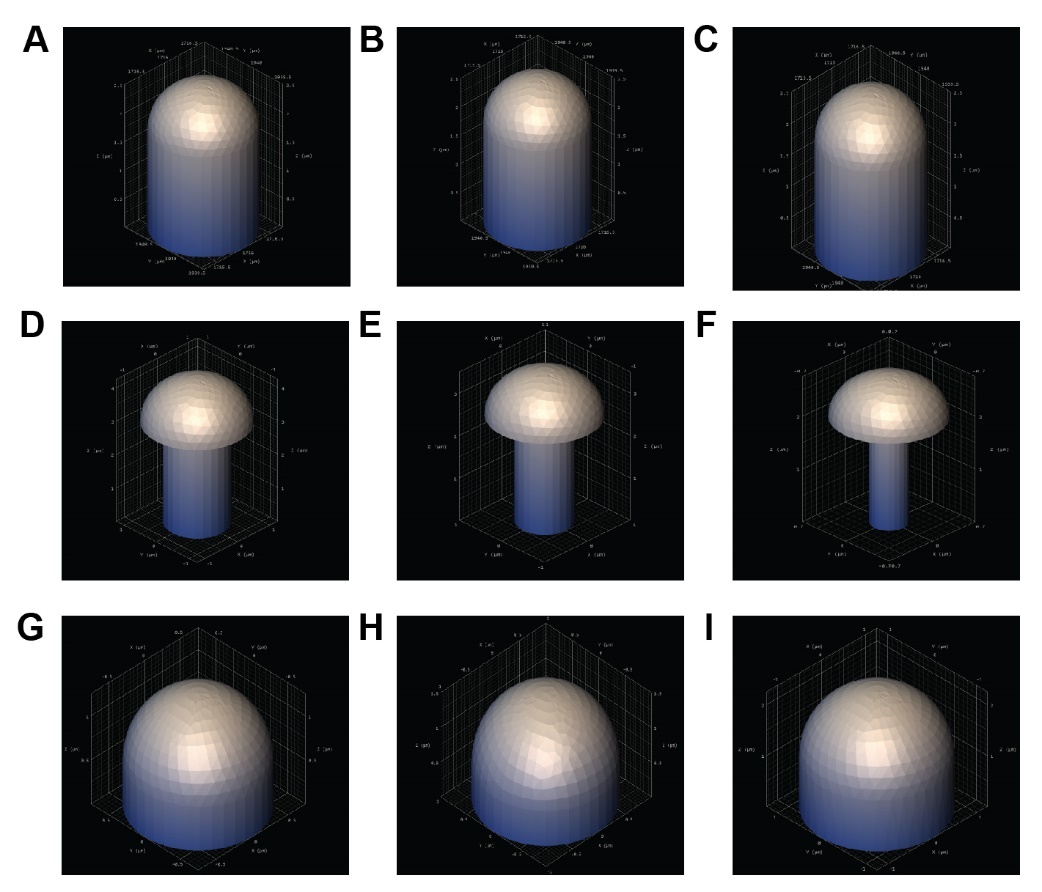


**Figure S1**. The design of the microstructures was obtained by using autoCAD software, and then the render has been completed in DeScribe software. Thin microstructures A) D_h_= 0.5 µm, D_n_= 0.5 µm, h=1.5 µm, B) D_h_= 1 µm, D_n_= 1 µm, h=2 µm; C) D_h_= 1.5 µm, D_n_= 1.5 µm, h=2.5 µm.

Mushroom-shaped microstructures D) D_h_= 1.5 µm, D_n_= 0.5 µm, h=1.5 µm E) D_h_= 2 µm, D_n_= 1 µm, h=2 µm F) D_h_ = 2.5 µm, D_n_ = 1.5 µm, h=2.5 µm.

Stubby microstructures G) D_h_ = 1.5 µm, D_n_ = 1.5 µm, h=1.5 µm H) D_h_ = 2 µm, D_n_ = 2 µm, h=2 µm I) D_h_ = 2.5 µm, D_n_ = 2.5 µm, h=2.5 µm. The sizes correspond to the **Table S1**.

**Supplementary Table S1**

Morphological parameters of artificial dendritic spines with varying densities and shapes

|  | Thin (D_h_=D_n_) | | | Mushroom (D_h_>D_n_) | | | Stubby | |  | | Density |
| --- | --- | --- | --- | --- | --- | --- | --- | --- | --- | --- | --- |
|  | **A** | **B** | **C** | **D** | **E** | **F** | **G** | **H** | **I** |  |  |
| Head Diameter (D_h_) | 0.5 | 1 | 1.5 | 1.5 | 2 | 2.5 | 1.5 | 2 | 2.5 | µm |  |
| Neck Diameter (D_n_) | 0.5 | 1 | 1.5 | 0.5 | 1 | 1.5 | 1.5 | 2 | 2.5 | µm |  |
| Spine Height (h) | 1.5 | 2 | 2.5 | 1.5 | 2 | 2.5 | 1.5 | 2 | 2.5 | µm |  |
| Pitch | 4 | | | 4 | | | 4 | | | µm | High |
| Pitch | 10 | | | 10 | | | 10 | | | µm | Medium |
| Pitch | 30 | | | 30 | | | 30 | | | µm | Low |

**Table S1.** Morphological parameters of fabricated microstructures categorized by shape (thin, mushroom-shaped, stubby) and density (high, medium, low). Head diameter (Dh), neck diameter (Dn), and spine height (h) are given in micrometers (µm). Density is indirectly represented by the pitch value (distance between spines): a smaller pitch corresponds to higher spine density.

**Supplementary Figure S2**

**
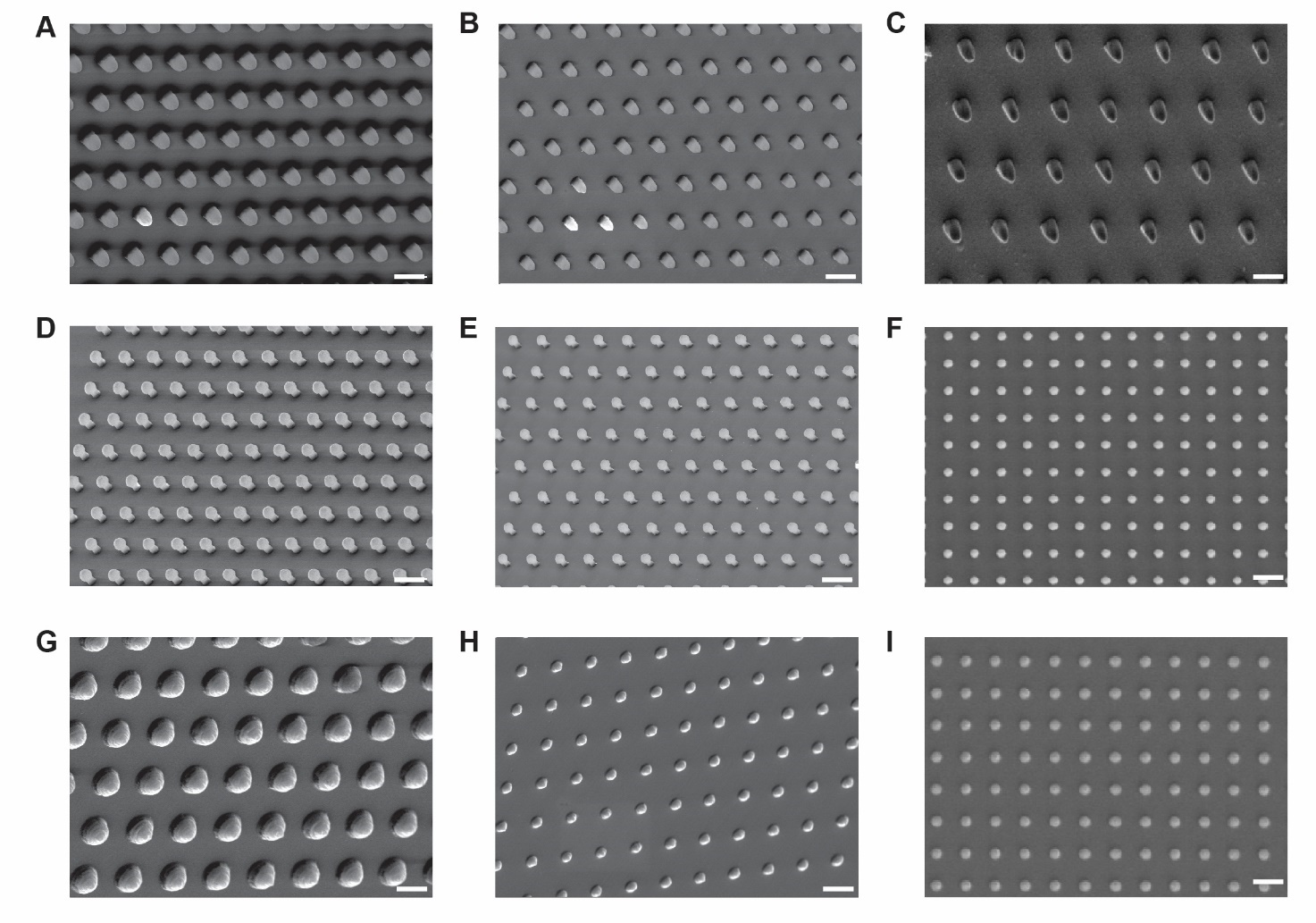
**

**Figure S2.** Scanning electron micrographs of high-density microstructures arrays with varying dimensions. The sizes of the microstructures correspond to those reported in **Table S1**. A), B) C) Thin microstructures, scale bar 4 µm. D), E) F) Mushroom-shaped microstructures, scale bar 2 µm; G) H) I) Stubby microstructures, scale bar 4 µm.

**Supplementary Figure S3**

**
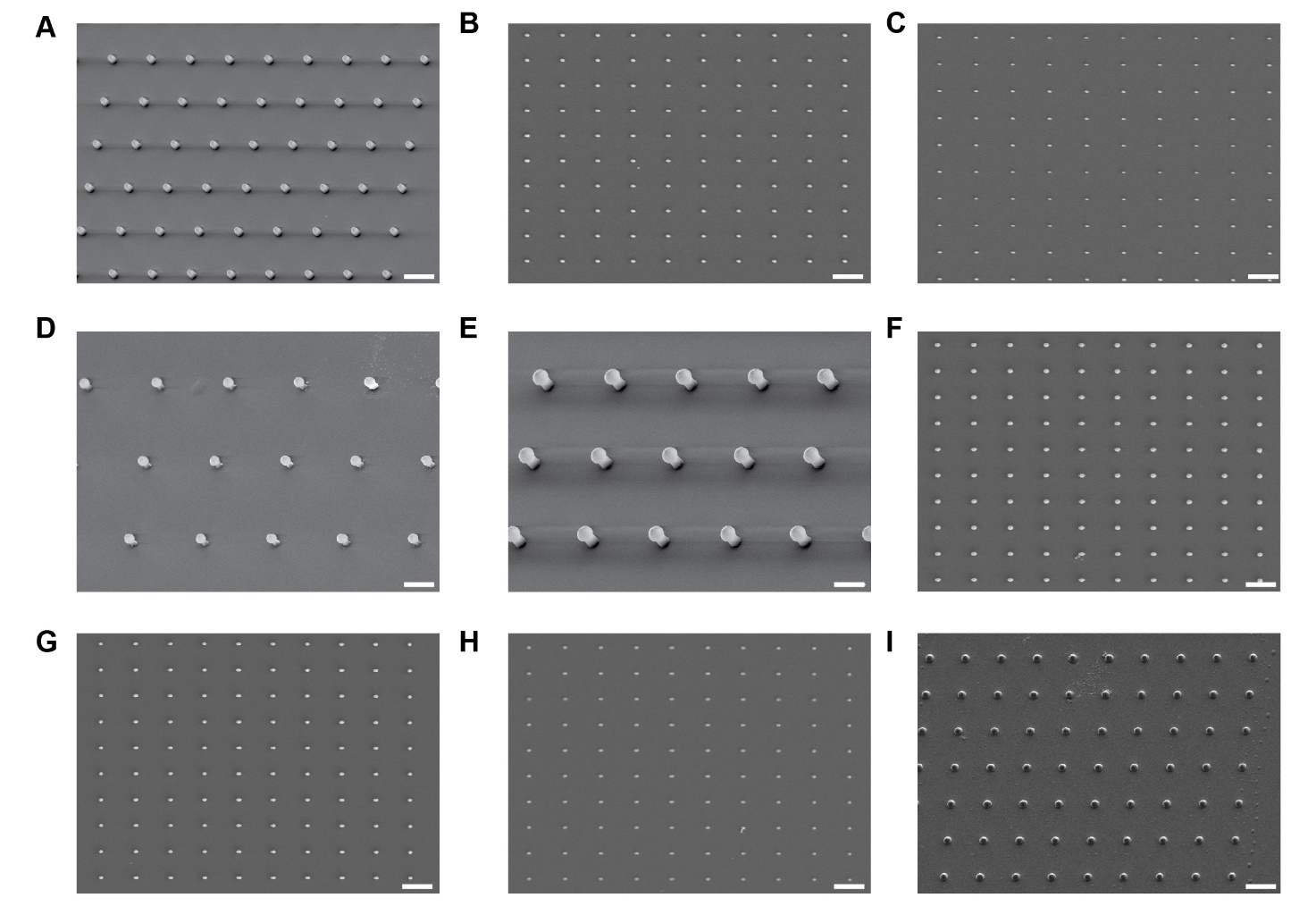
**

**Figure S3.** Scanning electron micrographs of medium-density microstructures arrays with varying dimensions. The sizes of the microstructures correspond to those reported in **Table S1**. A), B) C) Thin microstructures, scale bar 10 µm. D), E) F) Mushroom-shaped microstructures, scale bar 2 µm and 10 µm; G) H) I) Stubby microstructures, scale bar 10 µm.

**Supplementary Figure S4**

**
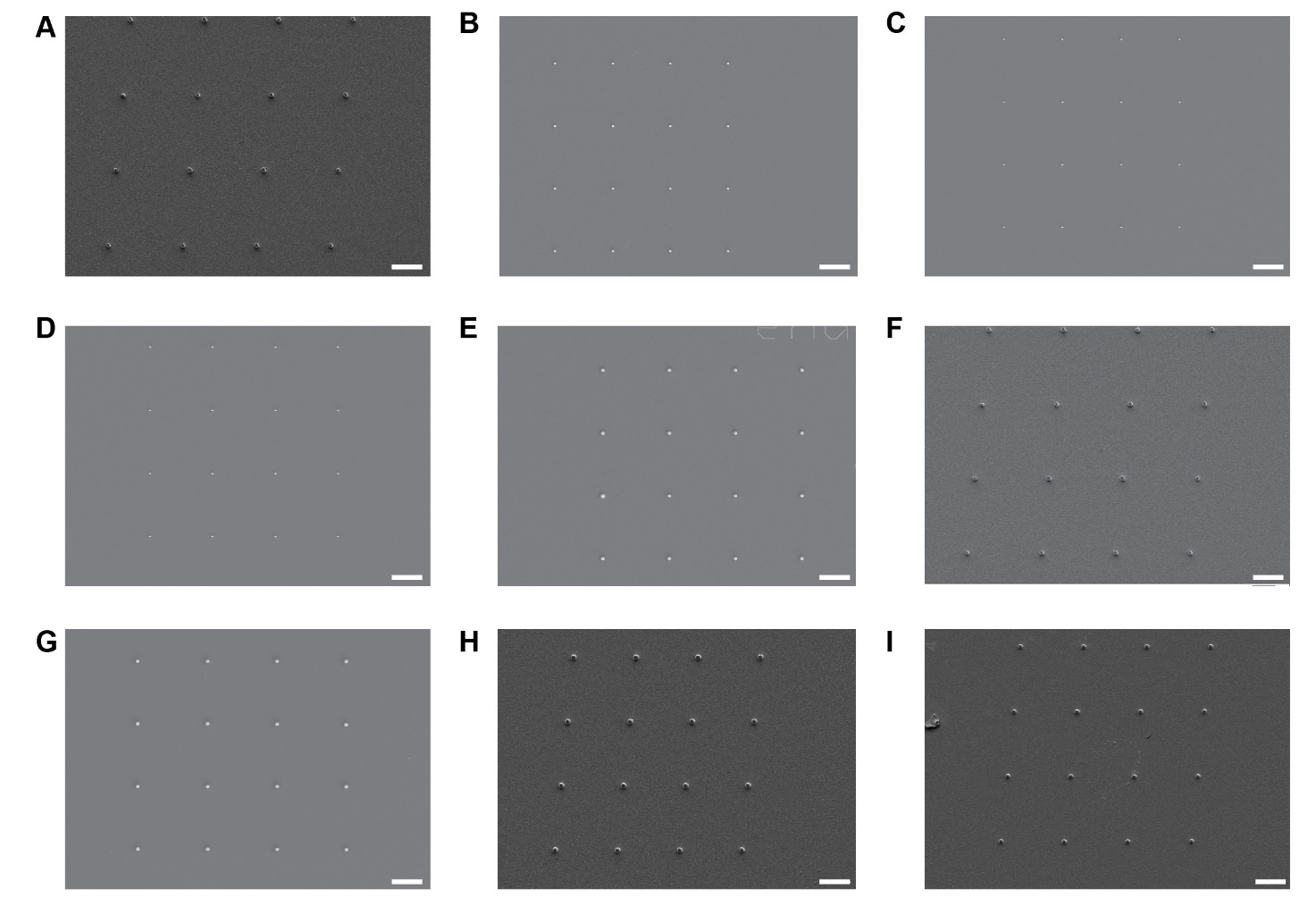
**

**Figure S4** Scanning electron micrographs of low-density microstructures arrays with varying dimensions. The sizes of the microstructures correspond to those reported in **Table S1**. A), B) C) Thin microstructures, scale bar 20 µm. D), E) F) Mushroom-shaped microstructures, scale bar 20 µm; G) H) I) Stubby microstructures, scale bar 20 µm.

**Supplementary Figure S5**


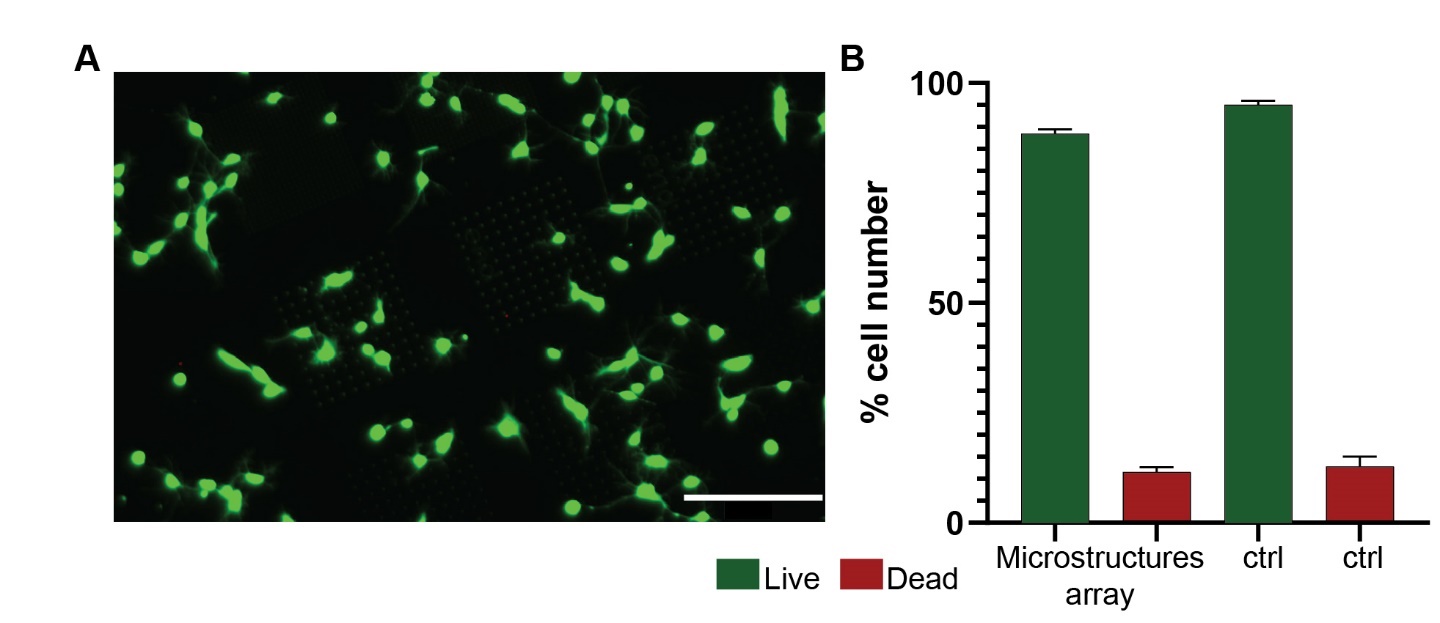


**Figure S5.** Live/dead cell viability assay after 24 hours of neuron culture on microstructure arrays.
A) Fluorescent microscopy image showing live cells (green) on the microstructure array, scale bar: 100 µm.B) Quantification of the percentage of live (green) and dead (red) cells on the microstructure arrays and control flat surfaces (ctrl). The results demonstrate a high viability of cells on the microstructures, comparable to control conditions, confirming the biocompatibility of the arrays.

**Supplementary Figure S6**


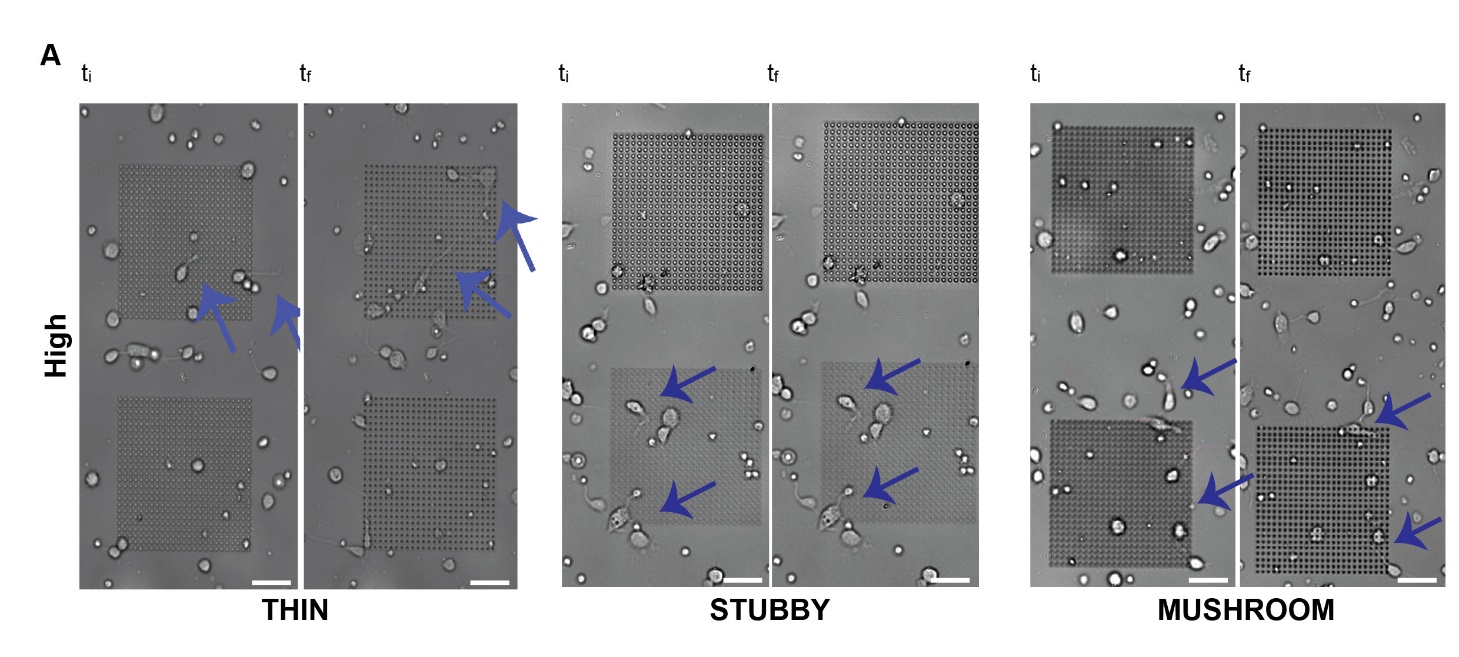


**Figure S6**. Live imaging of primary neurons after 24 hours on high-density microstructures arrays (P4). Selected frames depict the initial time point (**tᵢ**) and final time point (**t_ᶠ_**). A) Thin microstructures, B) Stubby microstructures, C) Mushroom-shaped microstructures. Blue arrows highlight neuronal interactions with the microstructures. Scale bar: 30 µm.

**Supplementary Figure S7**


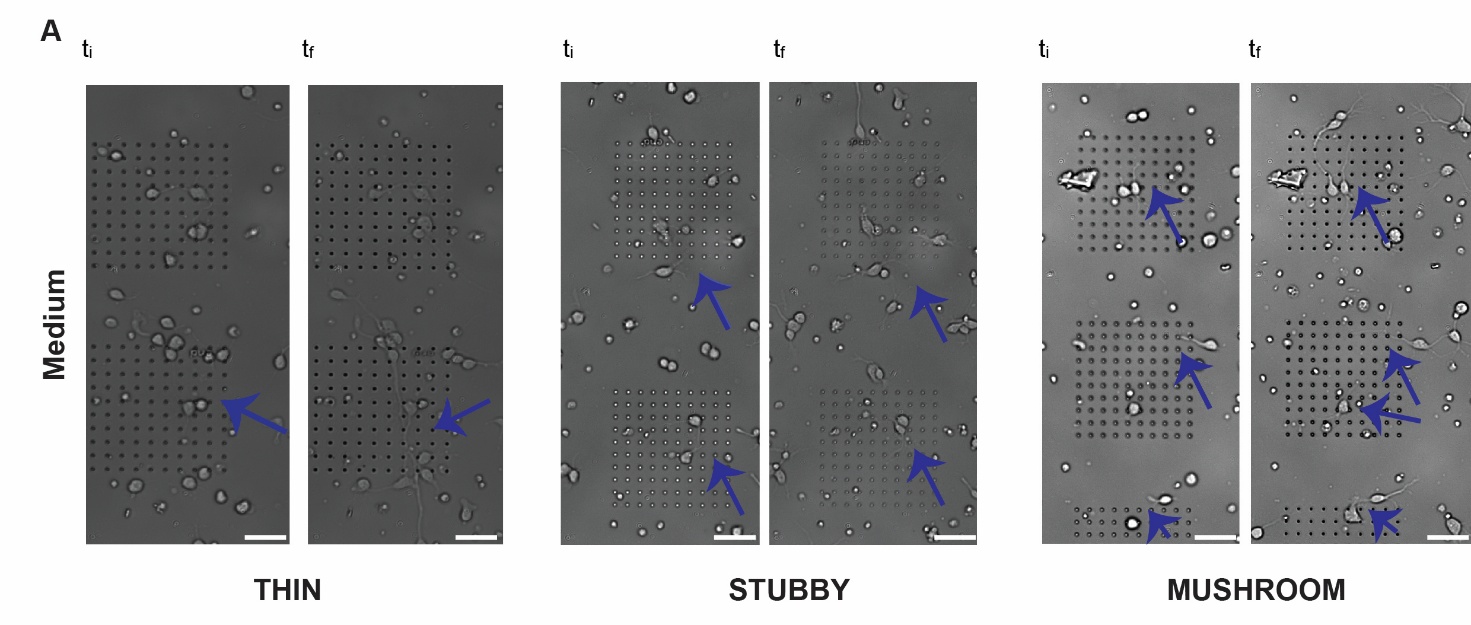


**Figure S7**. Live imaging of primary neurons after 24 hours on medium-density microstructures arrays (P10). Selected frames depict the initial time point (**tᵢ**) and final time point (**t_ᶠ_**). A) Thin microstructures, B) Stubby microstructures, C) Mushroom-shaped microstructures. Blue arrows highlight neuronal interactions with the microstructures. Scale bar: 30 µm.

**Supplementary Figure S8**


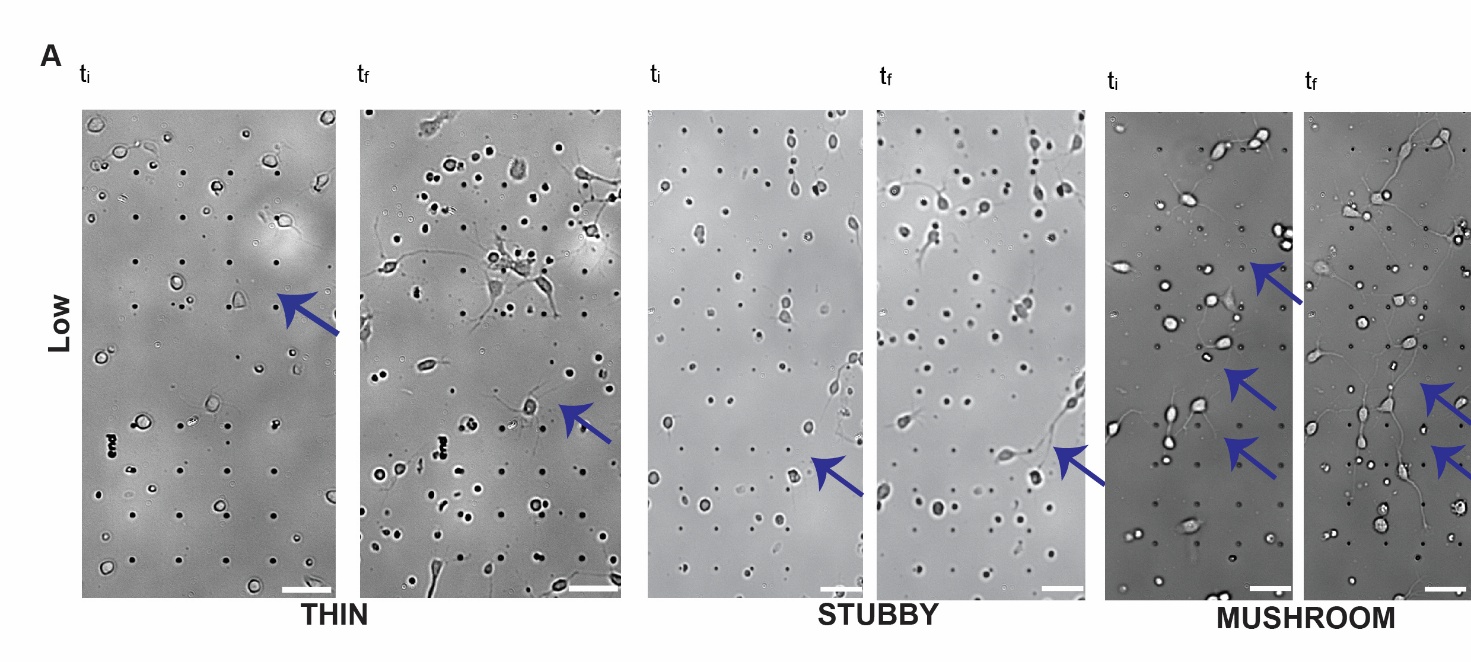


**Figure S8**. Live imaging of primary neurons after 24 hours on low-density microstructure arrays (P30). Selected frames depict the initial time point (**tᵢ**) and final time point (**t_ᶠ_**). A) Thin microstructures, B) Stubby microstructures, C) Mushroom-shaped microstructures. Blue arrows highlight neuronal interactions with the microstructures. Scale bar: 30 µm.

**Supplementary Figure S9**


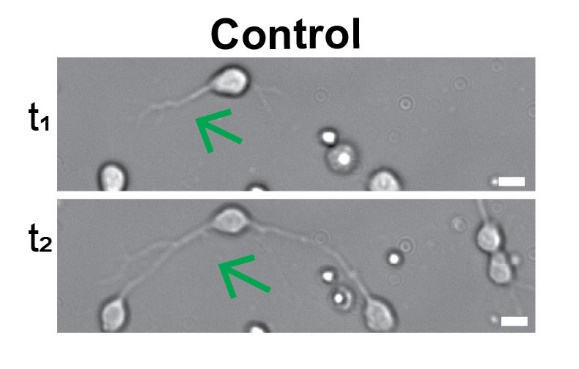


**Figure S9.** Live imaging of primary neurons after 24 hours on control flat substrate at two time points (t_1_ and t_2_). Scale bar 10 µm.

**Supplementary Figure S10**

**
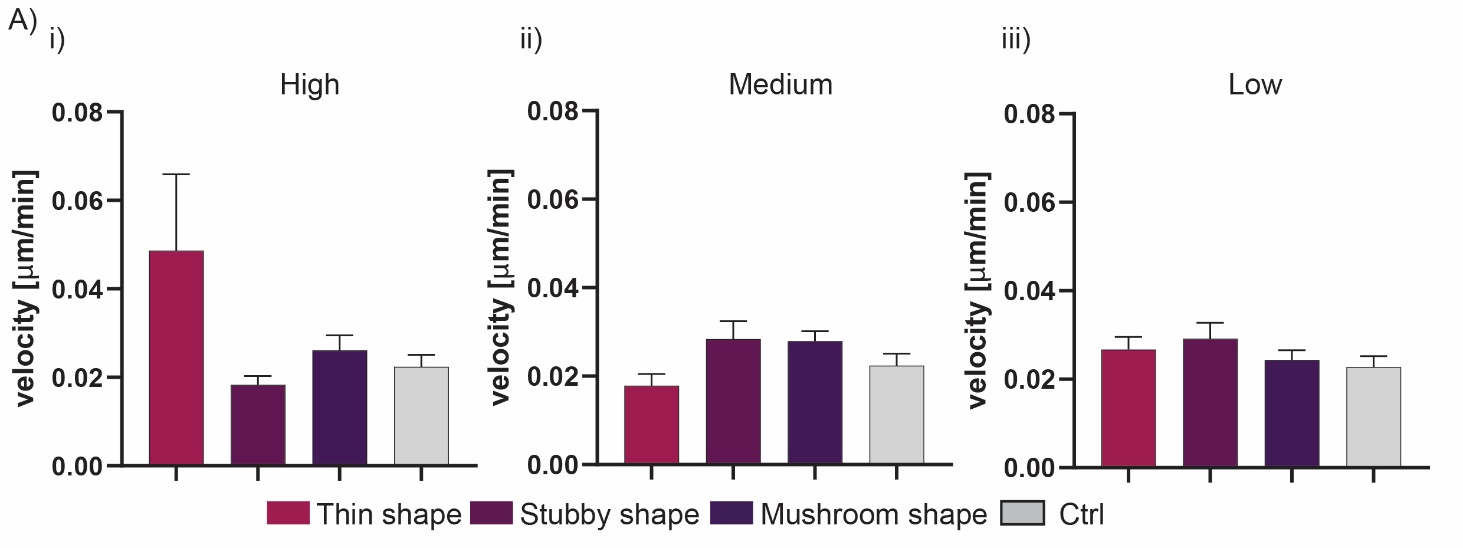
**

**Figure S10.** Average neurite velocity calculated to assess how pillar density and shape influence neurite movement. Higher velocities were observed in thin microstructures within A) high-density arrays, while B) medium- and C) low-density arrays showed similar velocity ranges.

**Supplementary Figure S11**


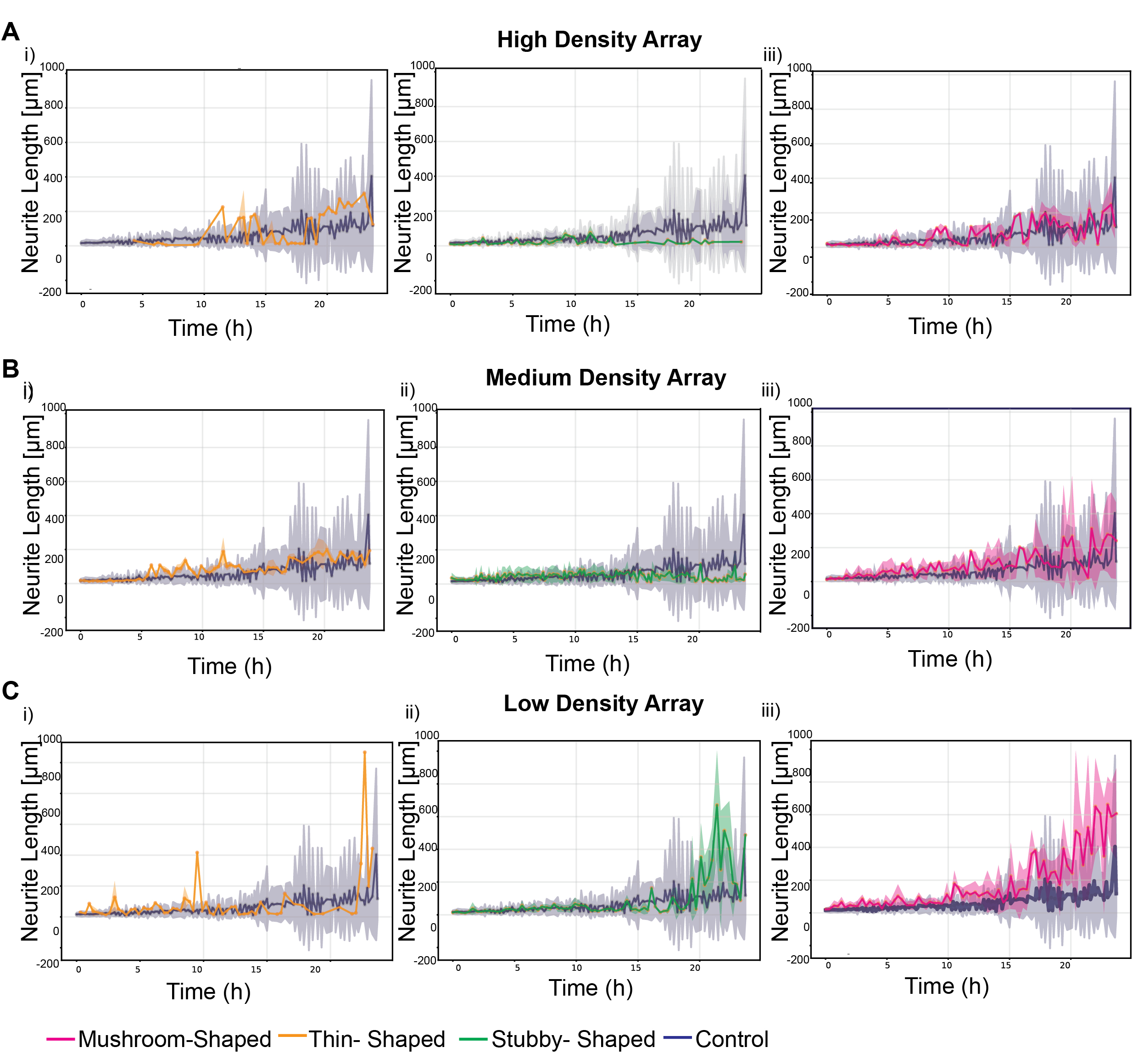


**Figure S11.** Retraction and growth phases monitored as of neurite length (µm). A) high-density arrays for i) thin microstructures, ii) stubby microstructures, iii) mushroom- shaped microstructures. B) medium-density arrays for i) thin microstructures, ii) stubby microstructures, iii) mushroom- shaped microstructures. C) low-density arrays for i) thin microstructures, ii) stubby microstructures, iii) mushroom- shaped microstructures. Color-coding for different shapes: mushroom (blue), thin (orange), stubby (green), and control (purple), standard deviation (shadow).

**Supplementary Figure S12**


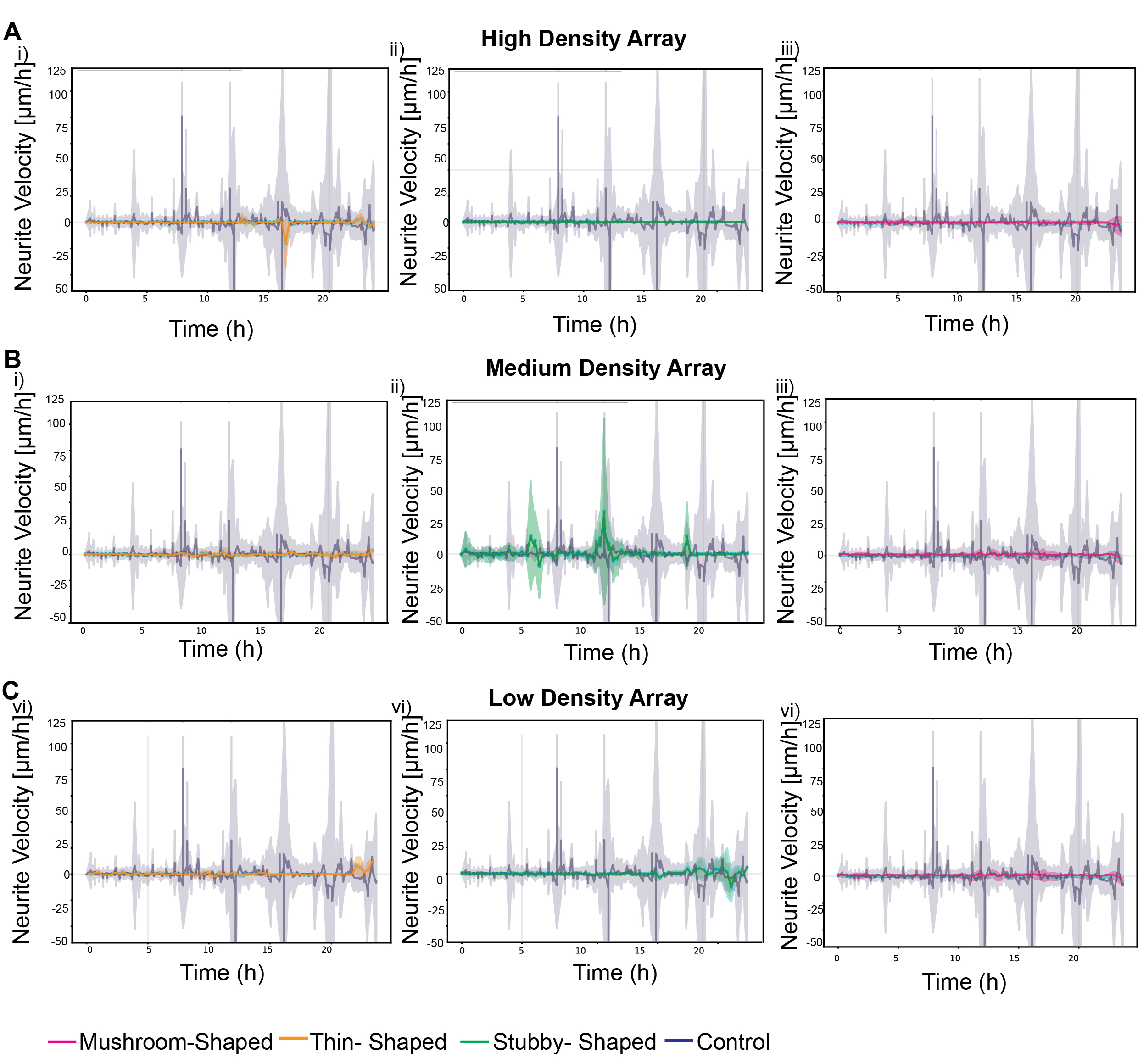


**Figure S12.** Retraction and growth phases monitored as average velocity (µm/h); A) high-density arrays for i) thin microstructures, ii) stubby microstructures, iii) mushroom- shaped microstructures. B) medium-density arrays for i) thin microstructures, ii) stubby microstructures, iii) mushroom- shaped microstructures. C) low-density arrays for i) thin microstructures, ii) stubby microstructures, iii) mushroom- shaped microstructures. Color-coding for different shapes: mushroom (blue), thin (orange), stubby (green), and control (purple), standard deviation (shadow)**.**

**Supplementary Figure S13**


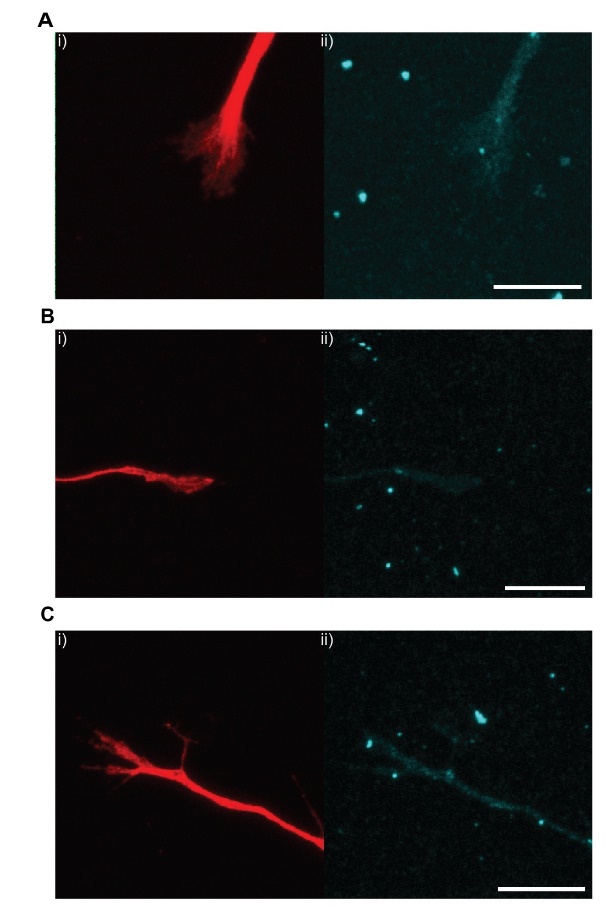


**Figure S13.** Control condition for growth cone configurations evaluated *via* immmunohistochemistry labelling for F-Actin (cyan) and β-III tubulin (red) highlighted the configuration as A) large growth cone, B) small growth cone, C) tree-like growth cone.

**Supplementary Figure S14**


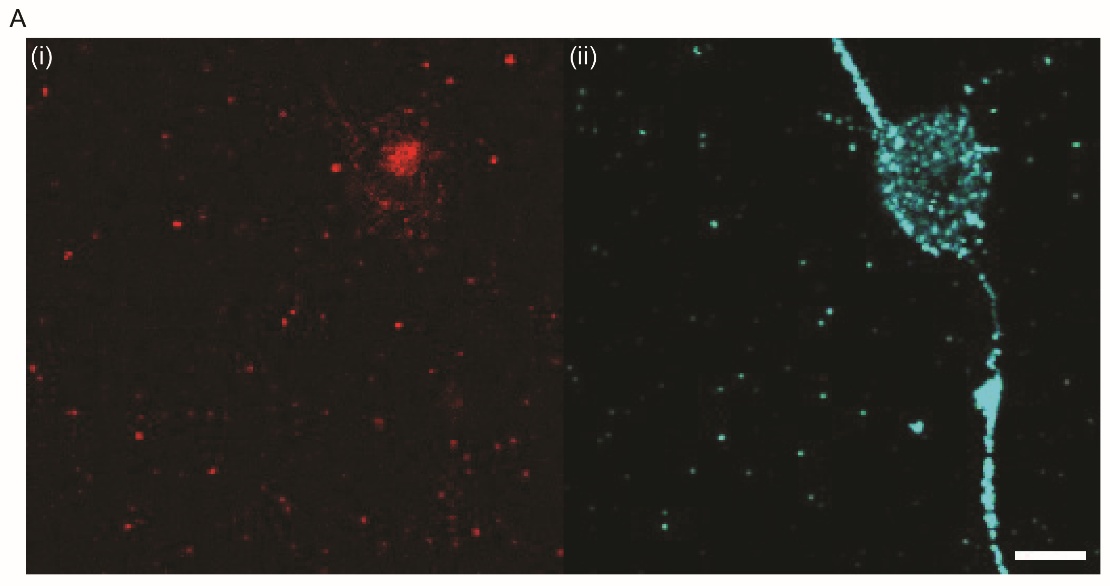


**Figure S14.** Neuronal cells cultured on control flat substrate with immmunohistochemistry labelling of i) paxillin (red, 647 nm) and ii) Integrin β1 (cyan, 546 nm), scale bar 5 μm.

**Supplementary Figure S15**


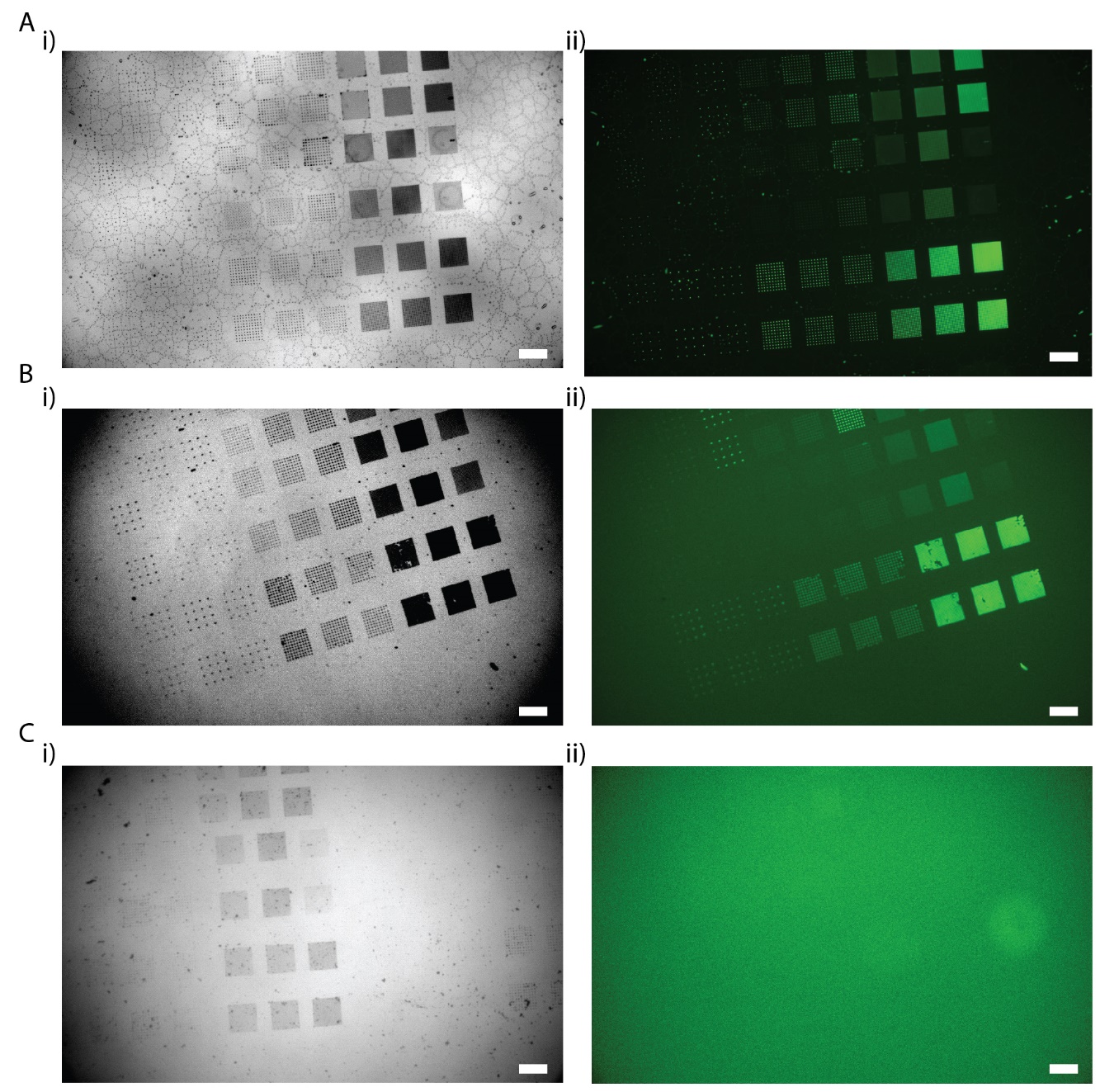


**Figure S15.** Auto-fluorescence quenching. A) control substrates without SBB treatment B) after SBB treatment and 2h of ethanol immersion. C) after SBB treatment and 2 hours in PBS solution; i) brightfield and ii) green channel (488 nm), scale bars 100 µm.

**Supplementary Figure S16**


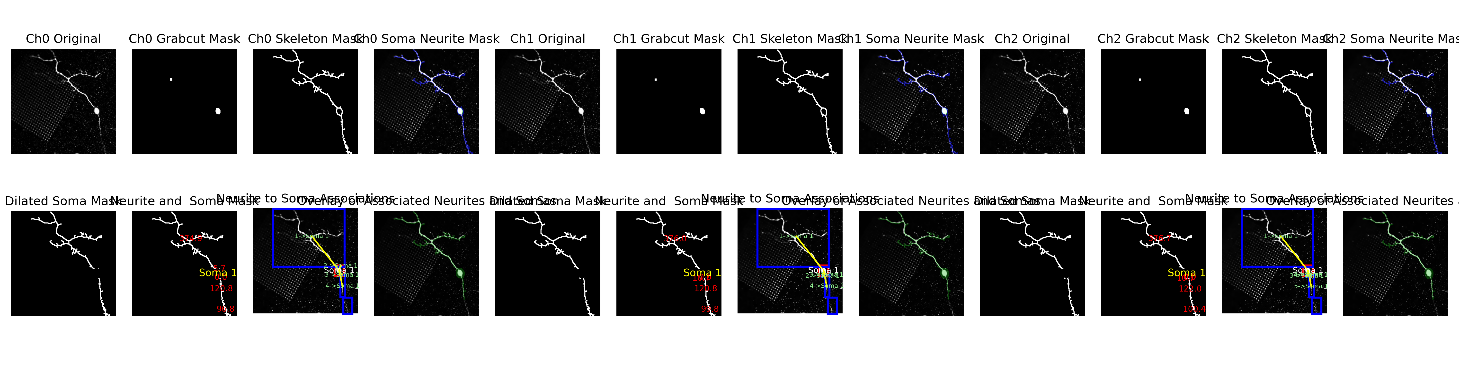


**Figure S16**. Representative image sequence illustrating the segmentation steps applied for the quantitative analysis of fluorescently labeled proteins.

**Supplementary Figure S17**


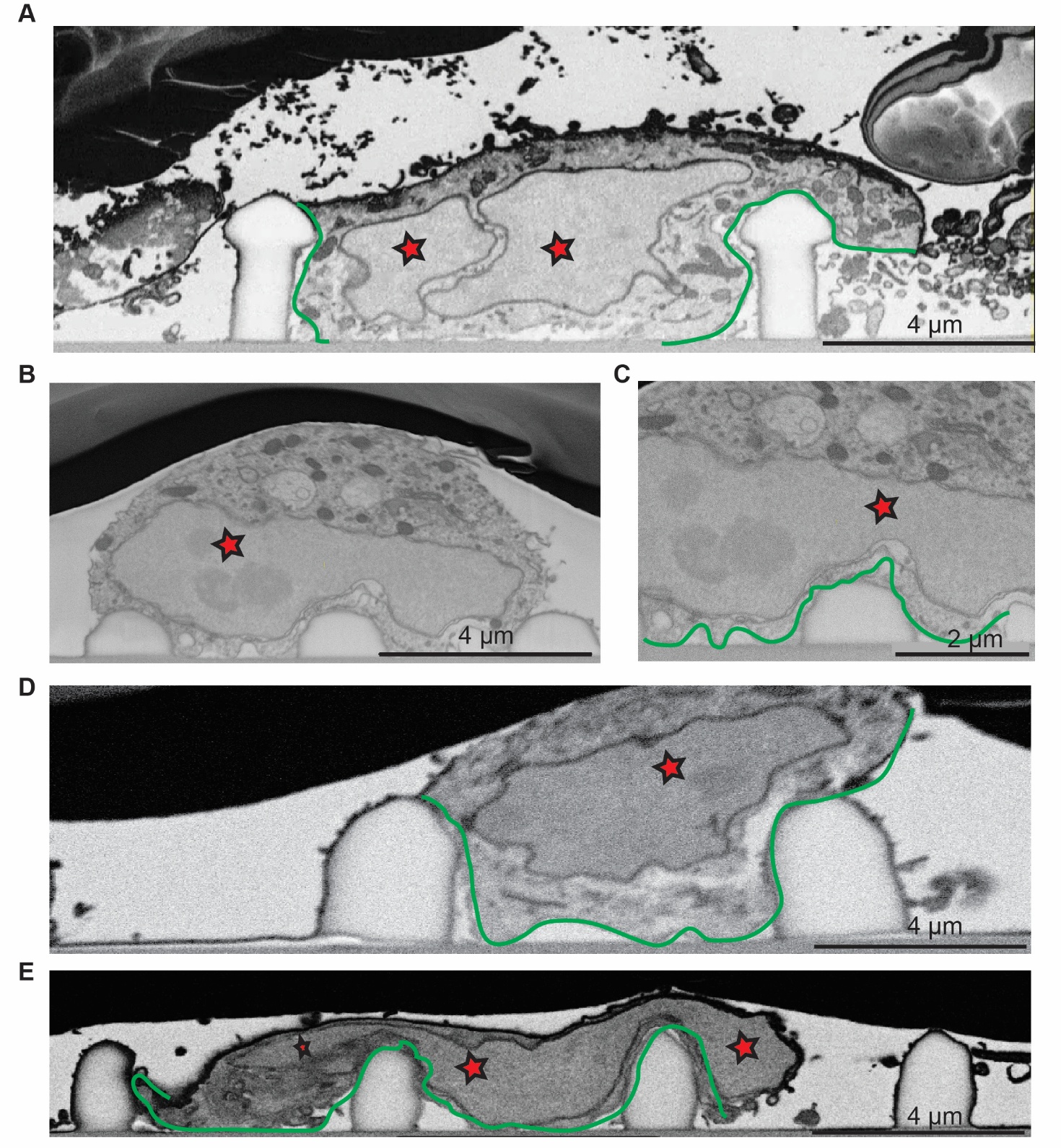


**Figure S17.** Scanning electron micrographs showing cross-sections of individual cells on microstructures. Samples were prepared by UTP (see **Experimental Section**). Red stars indicate the nucleus, while green lines highlight membrane invaginations. A) Mushroom-shaped microstructures, B) Stubby microstructures, C) High magnification of cells cultured on stubby microstructures, and D) E) thin microstructures.

**Supplementary Figure S18**


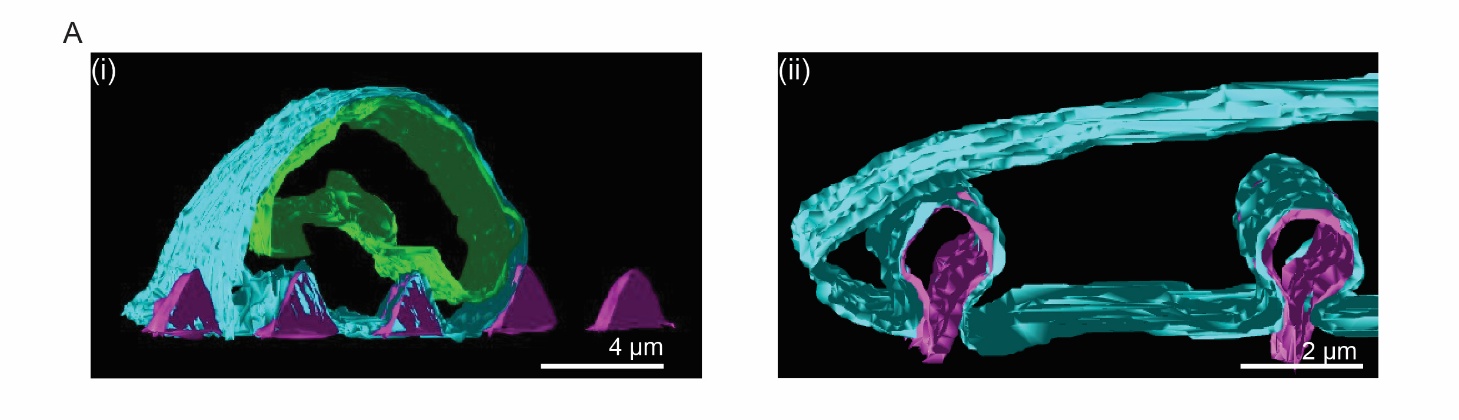


**Figure S18.** 3D reconstruction from sequential SEM-FIB of i) cell body and ii) neurites around microstructures.

**Supplementary Figure S19**


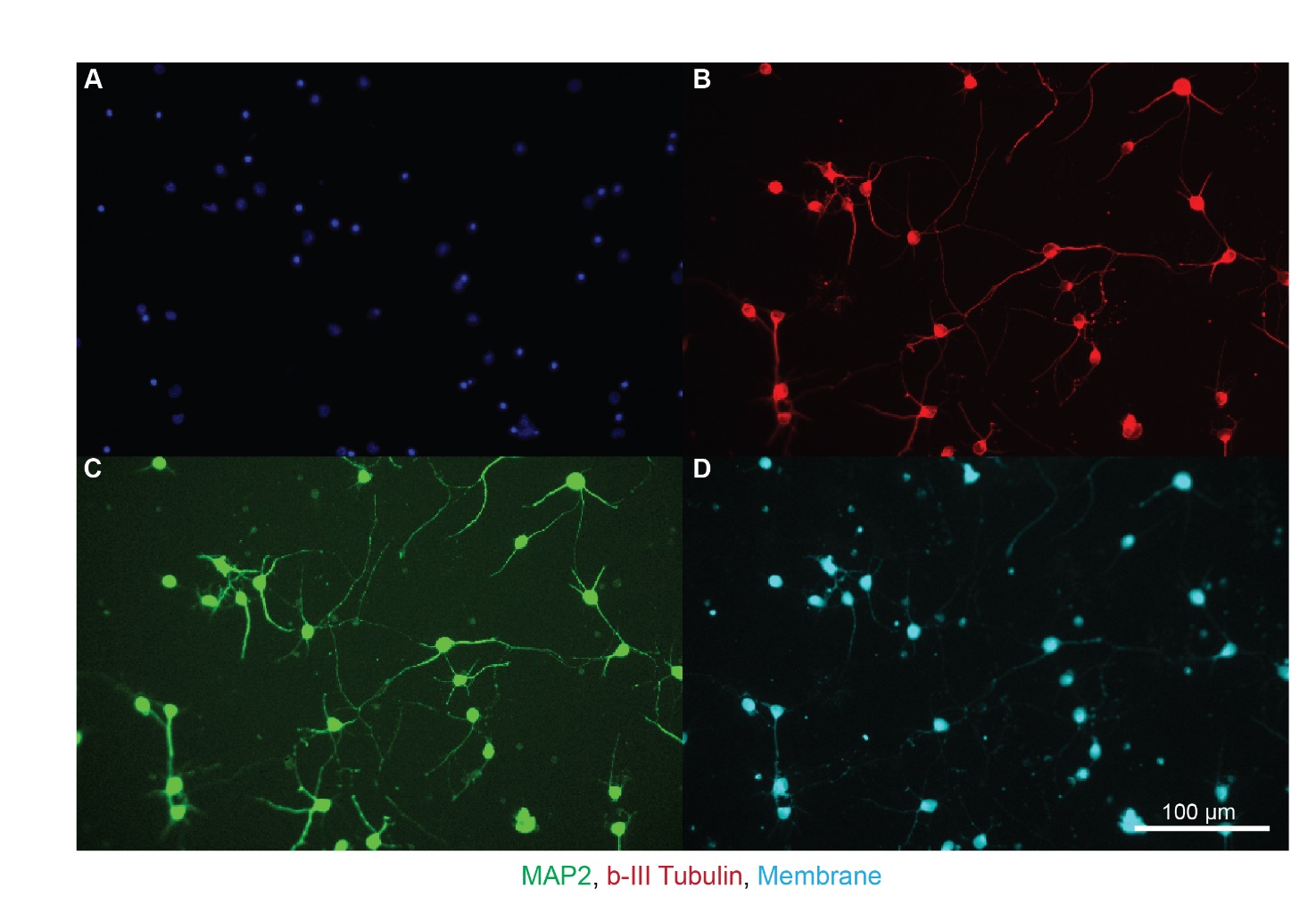


**Figure S19.** Fluorescence micrograph of neuronal cells cultured on control substrate and labelled with A) Hoechts for nuclei (blue, 361 nm), B) β-III- tubulin for microtubules (red, 647 nm), C) MAP2 microtubules (green,488 nm D) membrane (cyan, 546 nm).

**Supplementary Figure S20**


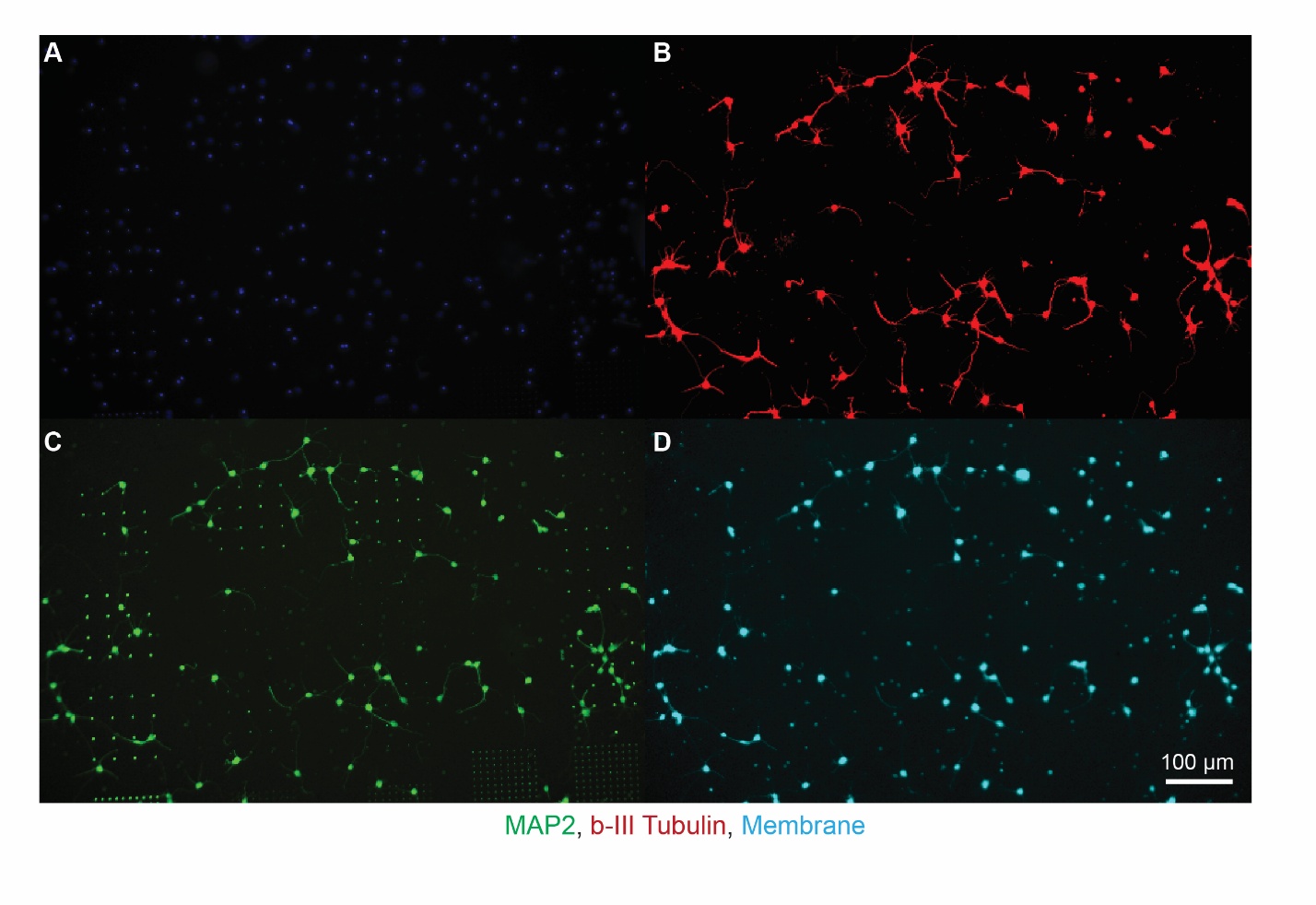


**Figure S20**. Fluorescence micrograph showing separate channels of neuronal cells cultured on an artificial spine substrate, labeled with A) Hoechst (blue, 361 nm) for nuclei, B) β-III-tubulin (red, 647 nm) for microtubules, C) MAP2 (green, 488 nm) for dendritic microtubules. Green dots represent the auto-fluorescent microstructures. Each microstructure shape (thin, stubby, mushroom) is shown at medium density (P10). D) Membrane staining (cyan, 546 nm).

**Supplementary Figure S21**


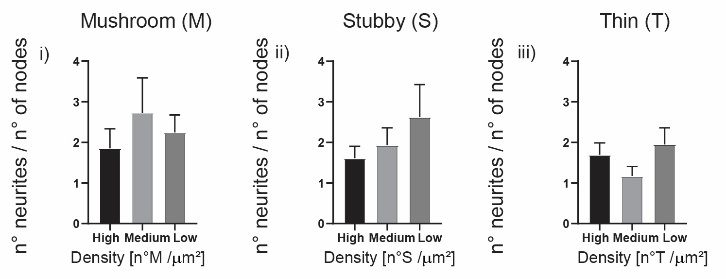


**Figure S21**. Branching analysis, number of neurites on number of nodes for : i) thin microstructures, ii) stubby microstructures, iii) mushroom-shaped microstructures.

**Supplementary Figure S22**


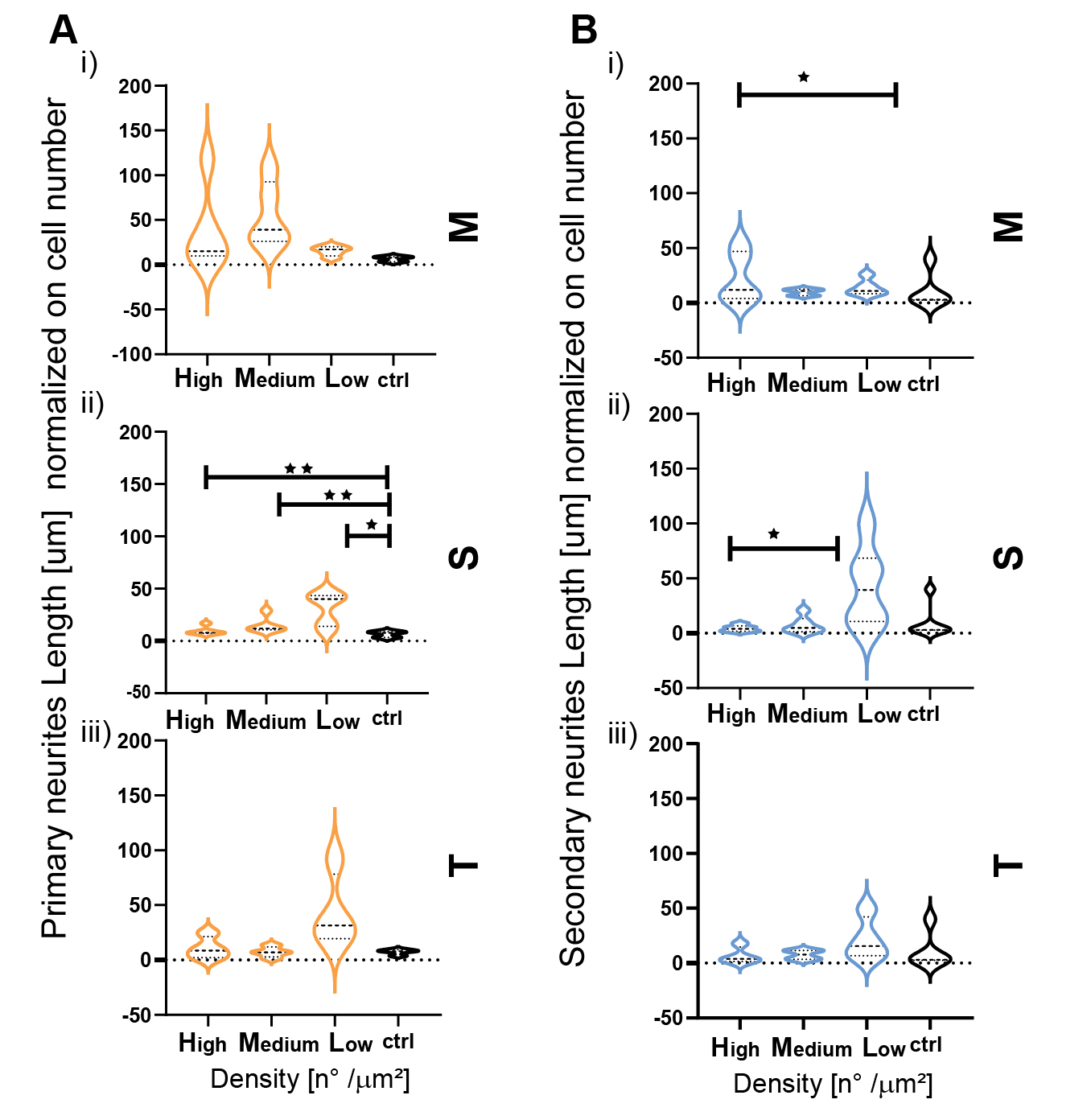


**Figure S22.** Elongation of the neurites normalized by on total cell number. A) Primary neurites elongation in the case of i) mushroom-shaped microstructures, ii) stubby microstructures, iii) thin microstructures. B) Secondary neurites elongation in the case of i) mushroom-shaped microstructures, ii) stubby microstructures, iii) thin microstructures.

**Supplementary Figure S23**

**
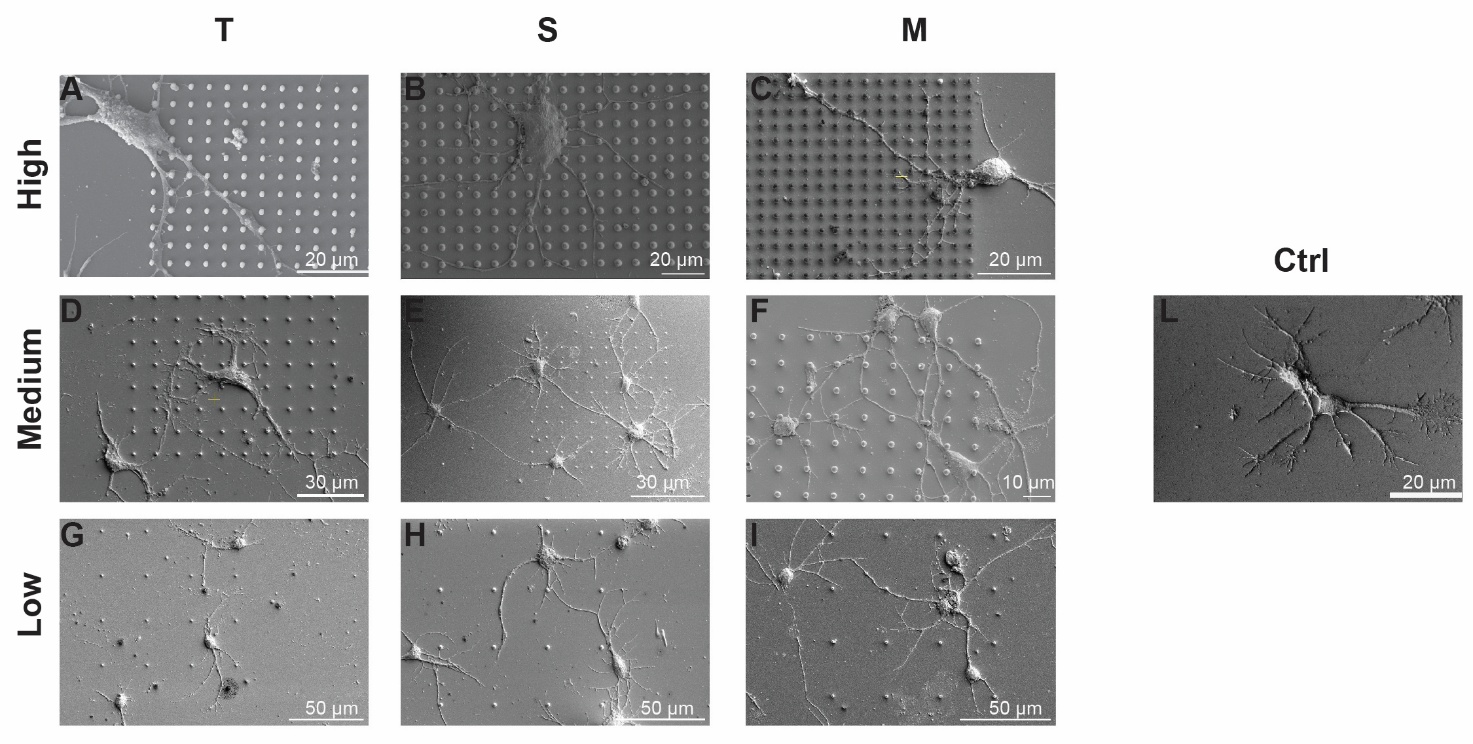
**

**Figure S23.** Scanning electron micrographs of neuronal cells fixed after 4 DIV on microstructures and control substrates, processed by CPD (**Experimental Section**). A-C) Neurons on high-density arrays, D-F) Neurons on medium-density arrays, and G-I) Neurons on low-density arrays. L) Neurons grown on control flat substrate.

**Supplementary Figure S24**

**
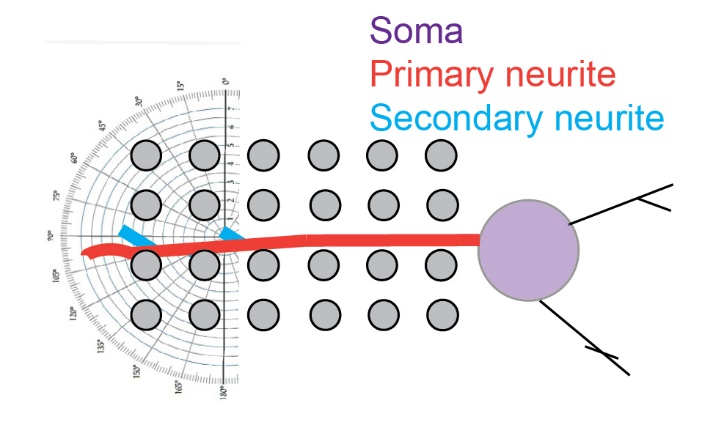
**

**Figure S24.** Calculation of guidance angle. The diagram illustrates the method for calculating the guidance angle of a neurite as it extends from the soma (purple circle) across a grid of microstructures (gray circles). The red line represents the neurite path, while the blue arc indicates the guidance angle. This angle is measured between the neurite's initial direction (before encountering microstructures) and its deflection as it navigates around or between microstructures. The guidance angle is calculated by determining the difference between the neurite's original path and its adjusted trajectory, with respect to the microstructure layout. This is typically measured using a polar coordinate system, as shown by the semicircular plot in the image.

**Supplementary Figure S25**


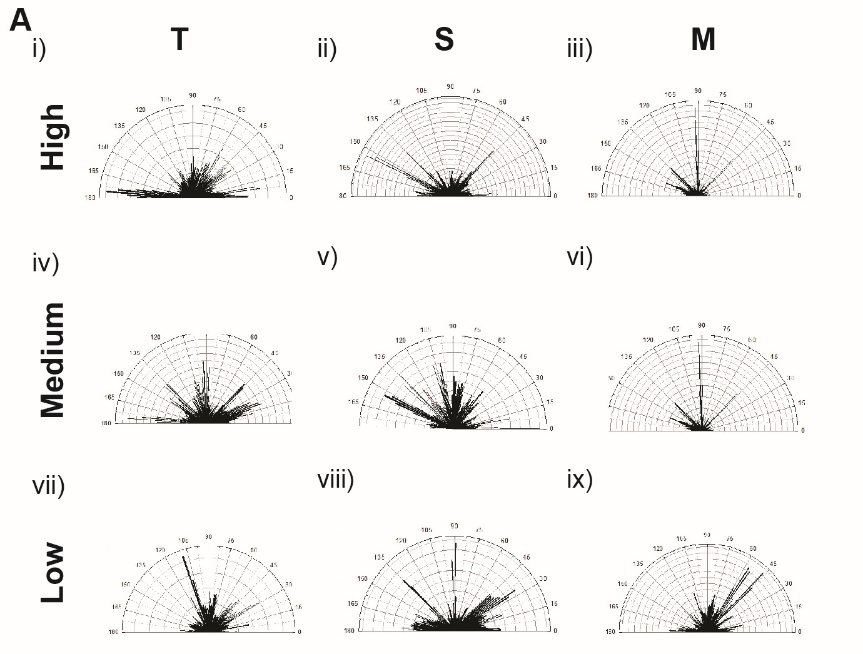


**Figure S25**. Directionality analysis, presented as polar plots (0-π), for each shape and density: high-density arrays (High) i) thin microstructures, ii) stubby microstructures, iii) mushroom-shaped microstructures; medium-density arrays (Medium) iv) thin microstructures, v) stubby microstructures, vi) mushroom-shaped microstructures and for low-density arrays (Low) vii) thin microstructures, viii) stubby microstructures, ix) mushroom-shaped microstructures.
